# Therapeutic Effects of Wnt5a-Overexpressing Bone Marrow Mesenchymal Stem Cells on Endothelial Barrier Repair in Acute Lung Injury

**DOI:** 10.3390/biom16091356

**Published:** 2026-09-17

**Authors:** Shiqi Li, Wanmei He, Manliang Guo, Xintong Yang, Mian Zeng

**Affiliations:** 1 Division of Pulmonary and Critical Care Medicine, The First Affiliated Hospital of Sun Yat-sen University, Guangzhou 510080, China; lishq77@163.com (S.L.);; 2 Institute of Respiratory Diseases, Sun Yat-sen University, Guangzhou 510080, China

**Keywords:** acute respiratory distress syndrome, bone marrow mesenchymal stem cell, endothelial barrier, Wnt5a

## Abstract

Background: Acute lung injury/acute respiratory distress syndrome (ALI/ARDS), a common complication of sepsis, is critically characterized by disruption of the alveolar–capillary barrier. The use of mesenchymal stem cells (MSCs) has emerged as a promising therapeutic strategy for ARDS owing to their potent paracrine effects. Wingless-type MMTV integration site family member 5A(Wnt5a) is a secreted protein with context-dependent effects on angiogenesis. This study aimed to investigate whether Wnt5a contributes to bone marrow-derived MSC (BMSC)-mediated repair of endothelial injury and whether Wnt5a-overexpressing BMSCs improve outcomes in an ALI animal model. Methods: Lipopolysaccharide (LPS) was used to induce endothelial cell (ECs) injury in vitro. EC proliferation, migration, tube formation and permeability, together with the expression of the junctional proteins zonula occludens-1 (ZO-1) and vascular endothelial cadherin (VE-cadherin) and the apoptosis-related proteins Bax and Bcl-2, were assessed after coculture with genetically modified BMSCs. In vivo, ALI was induced in mice by intraperitoneal administration of LPS. Lung histopathology, the lung wet-to-dry weight ratio, cytokine concentrations in bronchoalveolar lavage fluid (BALF) and serum, Evans blue extravasation, and the expression of junctional and apoptosis-related proteins were evaluated. PI3K/AKT signaling was examined as a potential pathway associated with the observed effects. Results: Compared with the vector-BMSCs, Wnt5a-overexpressing BMSCs increased the proliferation, migration, and tube formation of LPS-injured ECs; reduced endothelial permeability; preserved junctional protein expression; and shifted apoptosis-related protein expression towards an anti-apoptotic profile. Wnt5a knockdown attenuated these responses. In vivo, treatment with Wnt5a-overexpressing BMSCs was associated with less histological lung injury, lower inflammatory cytokine concentrations and a similar shift in apoptosis-related protein expression after LPS challenge. These protective effects were accompanied by enhanced PI3K/AKT signaling. Conclusions: Wnt5a overexpression optimized the protective effects of BMSCs against ALI in vivo and protected against LPS-induced EC barrier dysfunction in vitro, accompanied by enhanced PI3K/AKT signaling.

## 1. Introduction

Sepsis is a life-threatening syndrome of organ dysfunction caused by a dysregulated host response to infection. Septic shock, a severe subset of sepsis characterized by circulatory and metabolic abnormalities, carries a particularly high risk of death [1]. Approximately 30% of patients with sepsis develop acute respiratory distress syndrome (ARDS), a major cause of respiratory failure in critically ill patients, with reported mortality rates of 30–40% [2,3]. Disruption of alveolar–capillary endothelial barrier integrity is central to ARDS pathophysiology. This barrier comprises alveolar epithelial and capillary endothelial cell layers separated by a basement membrane and supports efficient gas exchange [4]. Acute lung injury (ALI) is a milder form of ARDS and similarly involves lung inflammation and impaired gas exchange [5]. Alveolar–capillary endothelial cells are metabolically active and are among the earliest and most vulnerable targets of injury in ARDS [6]. Under physiological conditions, these cells regulate vascular tone and permeability, mediate nutrient transport, inhibit thrombosis, limit blood cell extravasation and suppress neutrophil adhesion [7]. Tight, adherens and gap junctions between adjacent endothelial cells maintain barrier integrity and fluid homeostasis [8]. Dysregulated inflammation disrupts these junctions and increases vascular permeability, suggesting that endothelial repair may be a relevant therapeutic target in ARDS.

Mesenchymal stem cells (MSCs) are multipotent cells with a capacity for self-renewal and multilineage differentiation. Initially isolated from bone marrow, MSCs have been identified in several tissues, including the umbilical cord [9]. MSCs can limit tissue injury, promote tissue repair and modulate immune responses [10]. Moreover, their relatively low immunogenicity also facilitates allogeneic transplantation. Notably, studies have demonstrated that intravenously administered MSCs preferentially localize to the pulmonary capillaries, a property that positions MSCs as a promising therapeutic strategy for ARDS [11].

Wnt5a is a secreted protein of the non-canonical Wnt signaling pathways in a receptor- and context-dependent manner, thereby regulating proliferation, migration, differentiation, and tissue development [12,13]. MSCs exert many of their therapeutic effects through secreted factors, and Wnt5a has been implicated in pulmonary vascular homeostasis. Wnt5a deficiency is associated with endothelial injury, microvascular rarefaction, and enhanced hypoxia-induced vascular remodeling in PAH models, whereas Wnt5a activation promoted angiogenic signaling via Ca^2+^-dependent non-canonical pathways [14,15,16,17]. MSC-derived exosomes have also been reported to increase Wnt5a expression, thereby reducing endothelial apoptosis and smooth muscle proliferation during hypoxia-induced pulmonary vascular remodeling [18]. In our previous study, ghrelin-preconditioned BMSCs provided greater protection against LPS-induced endothelial injury; this effect was accompanied by increased Wnt5a expression and AKT activation [19]. These observations prompted us to examine whether Wnt5a expression modifies the effects of BMSCs on alveolar endothelial cell injury in ARDS.

## 2. Materials and Methods

### 2.1. Animal Ethics

The study was approved by the Ethics Committee of the First Affiliated hospital, Sun Yat-sen University (ethical approval number: [2024]206).

### 2.2. BMSC Transduction and Culture

BMSCs were isolated from the bone marrow of male Sprague-Dawley (SD) rats obtained from the Animal Experimental Center of Sun Yat-sen University (Guangzhou, China) and cultured in low-glucose Dulbecco’s-Modified Eagle’s Medium (DMEM, Gibco, Grand Island, NE, USA, Cat# C11995500BT) supplemented with 10% fetal bovine serum (Procell, Wuhan, China, Cat# 164210) and 1% penicillin–streptomycin (Gibco, Grand Island, NE, USA, Cat# 15140122)under the standard conditions at 37 °C with 5% CO_2_. At passage 3, surface markers were analyzed by flow cytometry, and adipogenesis, osteogenesis and chondrogenesis differentiation assays were performed to confirm BMSC identity. Preliminary optimization identified a multiplicity of infection (MOI) of 125 as providing efficient transduction with acceptable cell viability. BMSCs were seeded in 6-well plates at a density of 2 × 10^5^/well and exposed to viral supernatant at an MOI of 125 with 20 μL of polybrene-plus. After 24 h, the medium was replaced with stem-cell medium (XRbio, Hangzhou, China, Cat# XRMSCPRO). Fluorescence was assessed 72 h after transduction, and transduced cells were selected with puromycin (2 μg/mL, Beyotime, Shanghai, China, Cat# ST551) for 72 h for subsequent experiments. For Wnt5a knockdown, siRNA transfection complexes were prepared according to the manufacturer’s instructions and added to BMSCs at a final siRNA concentration of 50 nM and changed to complete medium after 6 h. Wnt5a expression was assessed by quantitative real-time PCR (qRT-PCR) and Western blotting.

### 2.3. Cell Culture

EA.hy926 human umbilical vein endothelial cells (ECs) were obtained from Zhong Qiao Xin Zhou Biotechnology (Shanghai, China) and cultured in high-glucose DMEM. Transwell chambers with a 0.4 μm pore size (BIOFIL, Guangzhou, China) were used for coculture. Briefly, BMSC^Vector^ or BMSC^Wnt5a^ cells were seeded in the upper chambers, and ECs were cultured in the lower chambers. LPS (150 μg/mL; Escherichia coli, O111:B4, Sigma-Aldrich, St. Louis, MO, USA, Cat# L2630) was used to induce ECs injury based on a previous study [20], followed by coculture with the indicated BMSCs for 24 h. Cells were divided into six groups: Control, LPS, LPS + BMSC^Vector^, LPS + BMSC^Wnt5a^, LPS + siNC-BMSC, and LPS + siWnt5a-BMSC.

### 2.4. 5-Ethynyl-20-deoxyuridine (EdU) Assay

After 24 h of coculture, ECs were incubated with EdU for 2 h, fixed in 4% paraformaldehyde (PFA) for 30 min, and permeabilized with 0.5% Triton-X 100 for 15 min. ECs were then incubated with click-reaction solution in the dark for 30 min. Nuclei were counterstained with for 10 min and imaged by fluorescence microscopy (Leica, Hesse, Germany).

### 2.5. Scratch Assay

ECs were seeded in 6-well plates and cultured to monolayer confluence. Then ECs were scratched straight with a 200 μL pipette tip and washed gently with PBS. Serum-free medium was added, and ECs were cocultured with different BMSCs for 24 h. The scratch photographs were taken under a microscope at 0 h and 24 h, and the healing rate was calculated according to the following formula: wound healing percentage = (scratch area at 0 h—scratch area at 24 h)/scratch area at 0 h × 100%.

### 2.6. Tube Formation Assay

Matrigel containing growth factors (ABW, Xiamen, China, Cat# 082704) was spread in 24-well plates (300 μL/well) and allowed to polymerize for 30 min at 37 °C with 5% CO_2_. ECs were seeded onto the Matrigel-coated 24-well plates (1 × 10^5^/500 μL), exposed to LPS and cocultured with the indicated BMSCs for 3 h. Transwell inserts were then removed, and tube formation was imaged by microscopy and quantified in three randomly selected fields per well.

### 2.7. Cell Immunofluorescence Staining

ECs were seeded in 24-well plates at a density of 5 × 10^4^ per well and grown to confluence. ECs were exposed to LPS and cocultured with different groups of BMSCs for 24 h. The medium was removed, followed by washing with PBS 3 times, fixing by pre-cooled methanol for 15 min, and blocking at 37 °C for 30 min. Cells were incubated overnight at 4 °C with antibodies against ZO-1 (1:50, Proteintech, Wuhan, China, Cat# 21773-1-AP) and VE-cadherin (1:200, Cell Signaling Technology, Beverly, MA, USA, Cat# 2500), followed by the appropriate fluorescent secondary antibodies. Images were acquired by confocal microscopy and quantified using ImageJ (v1.53t, National Institutes of Health, Bethesda, MD, USA).

### 2.8. Cell Permeability Assay

ECs were seeded in the transwell inserts at a density of 5 × 10^5^ per well, exposed to LPS and cocultured with the indicated BMSCs for 24 h. The medium in the upper chambers was replaced with medium containing horseradish peroxidase-conjugated streptavidin (Solarbio, Beijing, China), and 1 mL of fresh medium was added to the lower chambers. After 20 min of incubation, 20 μL of medium from the lower chambers was transferred to a new 96-well plate and incubated with 50 μL of TMB substrate at room temperature for 30 min, followed by the addition of 25 μL of stop solution. Absorbance was finally measured at 450 nm using a microplate reader.

### 2.9. Preparing Experimental Animals

Specific pathogen-free (SPF) male C57BL/6 male mice were obtained from the Animal Experimental Center of the First Affiliated Hospital of Sun Yat-sen University. The ALI animal model was established by intraperitoneal injection of LPS (10 mg/kg). Mice were randomly divided into four experimental groups (*n* = 12 per group): (1) a control group that received 150 μL PBS via tail-vein injection; (2) an LPS group that received 150 μL PBS via tail-vein injection; (3) an LPS + BMSC^Vector^ group that received 1 × 10^6^ BMSCs in 150 μL PBS; (4) an LPS + BMSC^Wnt5a^ group treated with 1 × 10^6^ in 150 μL PBS. All cell suspensions were delivered through tail-vein injection. After 24 h of LPS challenge, mice were euthanized by isoflurane inhalation and samples were collected for subsequent analysis.

### 2.10. Lung Wet-to-Dry Weight Ratio

The right upper lung lobe was excised, rinsed gently with PBS to remove surface blood and blotted dry. The wet weight was recorded using a precision balance. Samples were then dried at 65 °C for 72 h and reweighed. The wet-to-dry weight ratio was calculated.

### 2.11. Histology

Lung tissues were fixed in 4% PFA, dehydrated, embedded and sectioned at 4 μm and stained with hematoxylin-eosin (H&E). Lung injury was scored for alveolar and interstitial inflammation, hemorrhage, pulmonary edema and atelectasis on a scale from 0 to 4 (0 = no injury, 1 = 25% visual field injury, 2 = 50% visual field injury, 3 = 75% visual field injury, and 4 = full visual field injury). Ten equally spaced fields per section were assessed in a blinded manner, and the component scores were summed to obtain the ALI score.

### 2.12. Evans Blue Assay (EB)

To evaluate pulmonary vascular permeability, EB dye (25 mg/kg, Sigma-Aldrich, St. Louis, MO, USA, Cat# E2129) in sterile saline was injected into tail veins 1 h before sacrifice. The mice were perfused intracardially with sterile saline, and the lungs were rapidly removed. Lung tissues (100 mg) were incubated in formamide at 65 °C for 72 h and centrifuged at 5000× *g*/min for 30 min. Supernatant absorbance was measured at 630 nm by a spectrophotometer.

### 2.13. Enzyme-Linked Immunosorbent Assay (ELISA)

The levels of tumor necrosis factor-α (TNF-α; Cat# JL10484), interleukin-1β (IL-1β; Cat# JL18442), and chemokine C-X-C-motif ligand 1 (CXCL1; Cat# JL20150) in serum and alveolar lavage fluid were measured using an ELISA kit (J&L Biological, Shanghai, China) according to the manufacturer’s instructions, and absorbance at 450 nm was detected by a spectrophotometer.

### 2.14. Tissue Immunofluorescence

After antigen retrieval and blocking, the lung tissue sections were incubated overnight at 4 °C with primary antibodies against VE-cadherin (1:1000, Cell Signaling Technology, Beverly, MA, USA, Cat# 2500), ZO-1 (1:2000, Proteintech, Wuhan, China, Cat# 21773-1-AP) and CD31 (1:2000, Servicebio, Wuhan, China, Cat# GB12063), followed by fluorescent secondary antibodies. The expressions of VE-cadherin and ZO-1 were observed via fluorescence microscopy and semi-quantified using ImageJ.

### 2.15. Western Blot Analysis

Total proteins from lung tissue and ECs were extracted using RIPA buffer supplemented with 1% PMSF and phosphatase inhibitor. Proteins were separated by 10% SDS-PAGE gel and transferred to PVDF (0.45 μm, Merck millipore, Germany, Cat# IPVH00010) membranes. Membranes were blocked for 15 min at room temperature and incubated overnight at 4 °C with primary antibodies against phospho-PI3K (1:1000, Abmart, Shanghai, China, Cat# T40116F), PI3K (1:1000, Abmart, Shanghai, China, Cat# T40115F), phospho-AKT (1:1000, Cell Signaling Technology, Beverly, MA, USA, Cat# 4060S), AKT (1:1000, Cell Signaling Technology, Beverly, MA, USA, Cat# 4691S), ZO-1 (1:1000, Proteintech, Wuhan, China, Cat# 21773-1-AP), VE-cadherin (1:1000, Cell Signaling Technology, Beverly, MA, USA, Cat# 2500), Bcl-2 (1:1000, Bioworld, Nanjing, China, Cat# BS1511), Bax (1:1000, Cell Signaling Technology, Beverly, MA, USA, Cat# 2772), Wnt5a (1:1000, Abclonal, Wuhan, China, Cat# A2133), GAPDH (1:10000, Proteintech, Wuhan, China, Cat# 160004-1-Ig), and Tubulin (1:1000, Proteintech, Wuhan, China, Cat# 10094-1-AP). After three washes with Tris-buffered saline containing Tween 20 (TBST), membranes were incubated with the respective enzyme-linked secondary antibodies for 1 h at room temperature. Bands were detected by enhanced chemiluminescence (Affinibody LifeScience, Wuhan, China, Cat# aiwb-006) and quantified using ImageJ.

### 2.16. Statistical Analysis

Data are presented as the means ± SEMs. Statistical analyses and graphical presentation were performed using GraphPad Prism 9.0 (San Diego, CA, USA). For pairwise comparisons, independent Student’s *t*-tests were applied, while multi-group analyses were conducted using one-way ANOVA followed by Tukey’s post hoc test. *p* < 0.05 was considered significant.

## 3. Results

### 3.1. Characteristic of BMSCs

**Figure 1:**
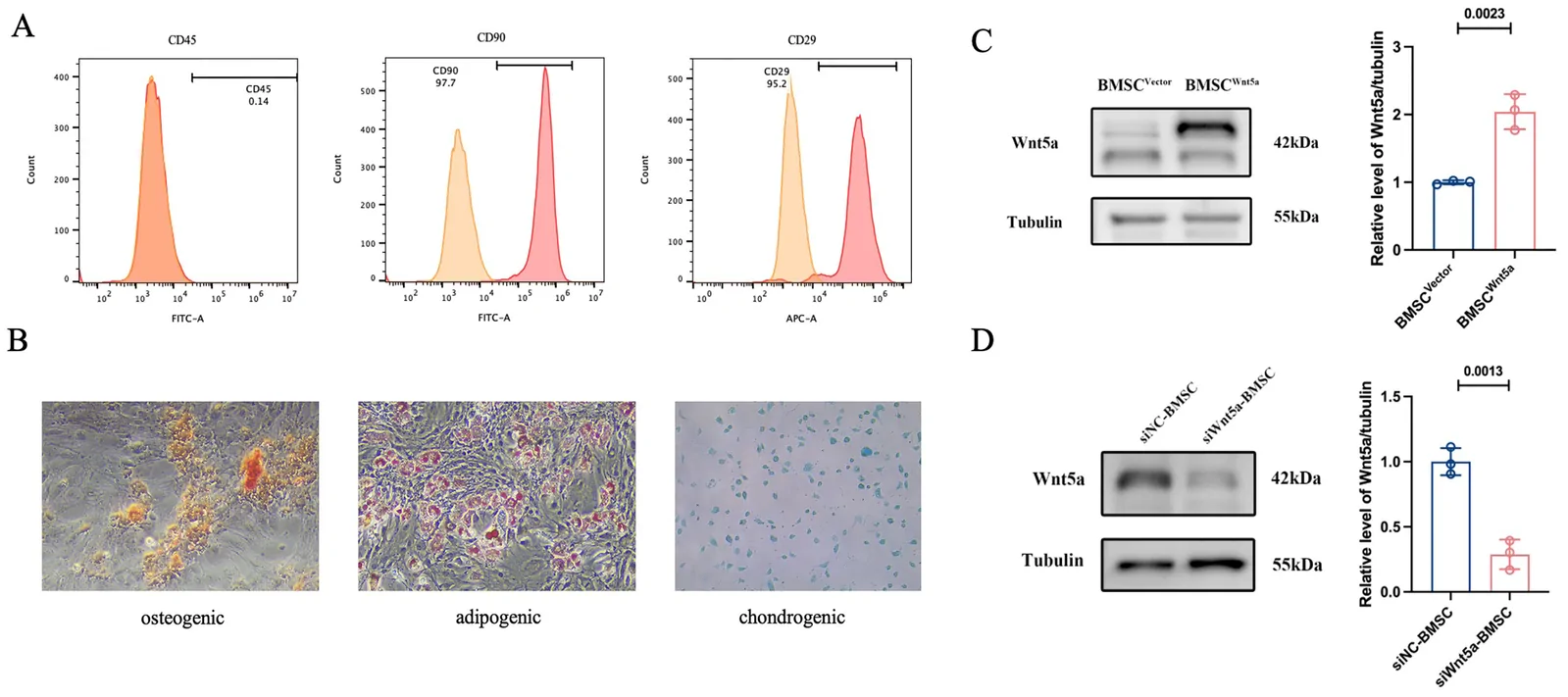
Isolation and characterization of rat BMSCs. (**A**) Flow cytometry analysis of BMSC surface markers, positive for CD29 and CD90, negative for CD45. (**B**) Osteogenic, adipogenic and chondrogenic differentiation assessed by Alizarin Red, Oil Red O, and Alcian Blue staining, respectively (100×). (**C**,**D**) Wnt5a expression in BMSCs were determined by Western blot analysis. Data are presented as means ± SEMs, *n* = 3. Compared with the corresponding control group. The blots were cropped and these uncropped images placed in Supplementary Material: Figures S1 and S2.

After the isolation and culture of rat BMSCs, cell surface markers were detected by flow cytometry. The results showed that BMSCs positively expressed CD29 and CD90 (>95% positive) but negatively expressed CD45 (<5% positive), such that they met the criteria for stem cell identification [21] (Figure 1A). Osteogenic, adipogenic, and chondrogenic differentiation was detected by Alizarin red, Oil red, and Alcian blue staining, respectively (Figure 1B). As shown in Figure 1C, the expression of Wnt5a was significantly increased in the BMSC^Wnt5a^ group, indicating that lentiviral-mediated overexpression efficiently enhanced Wnt5a expression in BMSCs. In contrast, Wnt5a expression was decreased in the siWnt5a-BMSC group compared with the siNC-BMSC group (Figure 1D).

### 3.2. Wnt5a-Overexpressing BMSCs Promoted Proliferation, Migration, and Tube Formation of LPS-Induced EC Injury In Vitro

**Figure 2:**
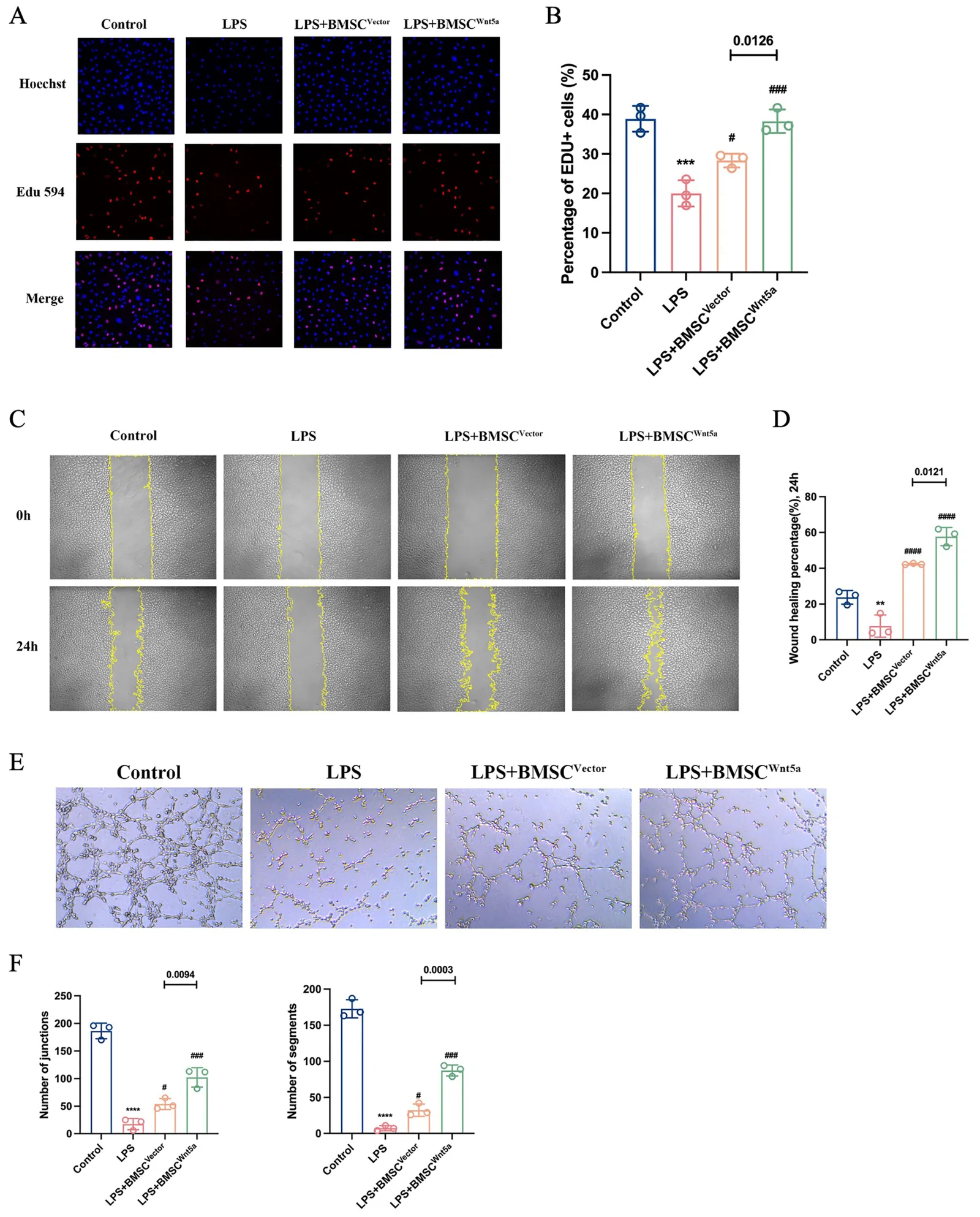
Wnt5a-overexpressing BMSCs promoted the proliferation, migration, and tube formation of LPS-induced EC injury in vitro. (**A**) EdU assay was used to evaluate the proliferation of LPS-induced EC injury, the blue color indicating nuclei and the red color indicating the proliferation-active cells (magnification: 200×). (**B**) Quantitative analysis was conducted by calculating the percentage of proliferation-active cells. (**C**) The scratch assay was used to assess the migration of ECs, and the representative images of the scratches at 0 h and 24 h are shown above (magnification: 50×). (**D**) Quantitative analysis of changes in the scratched area was performed using ImageJ. (**E**) Tube-formation assays were used to detect the angiogenic capacity of injured ECs. (**F**) Quantitative analysis indicated that coculture with BMSC^Wnt5a^ cells significantly promoted the tube formation of injured ECs compared with that in the BMSC^Vector^ coculture group. Data are presented as means ± SEMs, *n* = 3. Compared with the control group, ** *p* < 0.01, *** *p* < 0.001, **** *p* < 0.0001; compared with the LPS group, # *p* < 0.05, ### *p* < 0.001, #### *p* < 0.0001.

**Figure 3:**
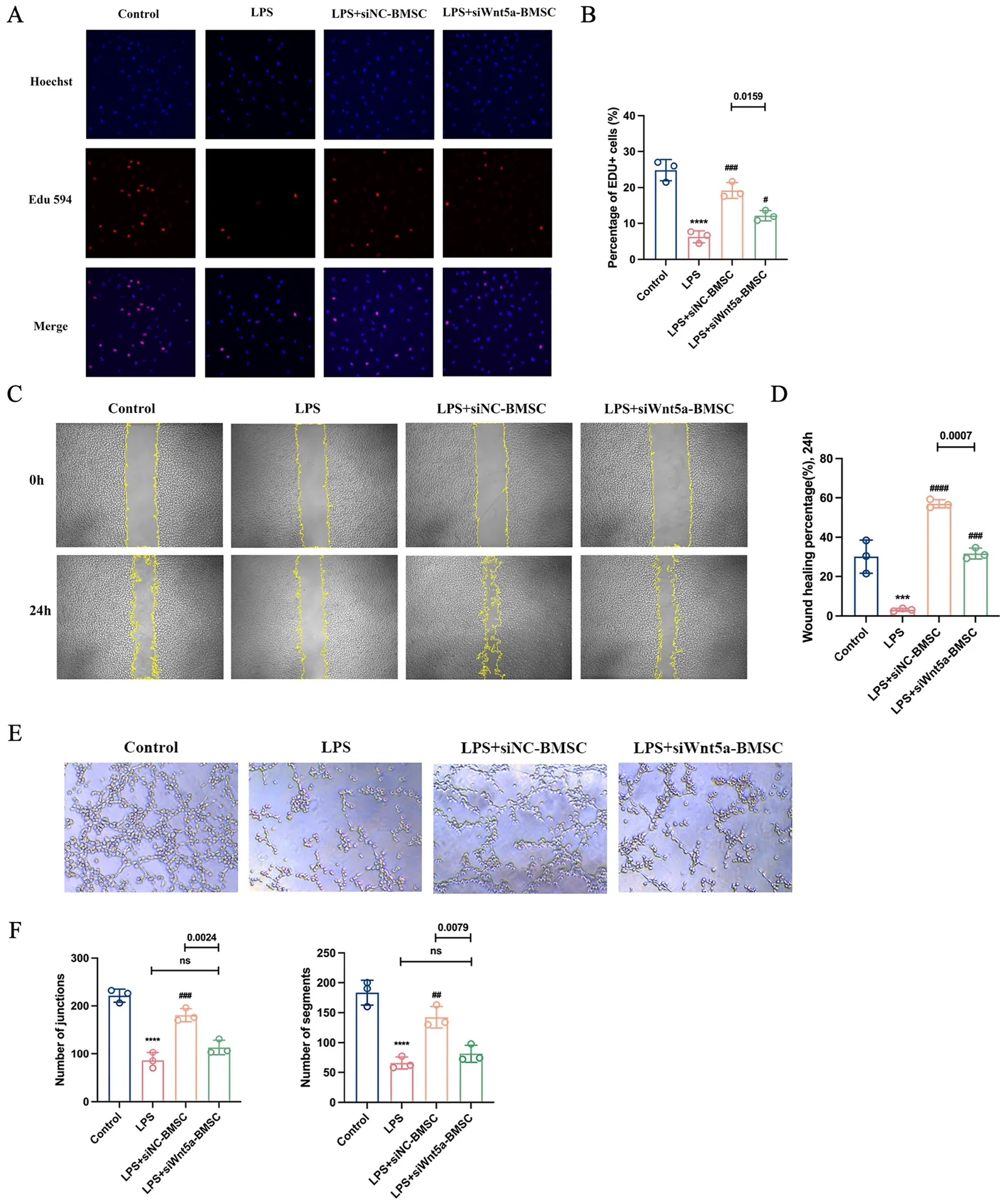
Wnt5a knockdown in BMSCs attenuated the proliferation, migration, and tube formation of LPS-induced EC injury in vitro. (**A**) EdU assay was used to measure the proliferation of LPS-induced EC injury, the blue color indicating nuclei and the red color indicating the proliferation-active cells (magnification: 200×). (**B**) Quantitative analysis was conducted by calculating the percentage of proliferation-active cells. (**C**) The scratch assay was used to assess the migration of ECs, and the representative images of the scratches at 0 h and 24 h are shown above (magnification: 50×). (**D**) Quantitative analysis of changes in the scratched area was performed using ImageJ, and the results showed that the migration ability of ECs was significantly decreased in the siWnt5a-BMSC coculture group compared with the siNC-BMSC group. (**E**) Tube-formation assays were used to detect the angiogenic capacity of LPS-induced ECs. (**F**) Quantitative analysis indicated that coculture with siWnt5a-BMSCs significantly decreased the tube formation of injured ECs compared with the siNC-BMSC group. Data are presented as means ± SEMs, *n* = 3. Compared with the control group, *** *p* < 0.001, **** *p* < 0.0001; compared with the LPS group, # *p* < 0.05, ## *p* < 0.01, ### *p* < 0.001, #### *p* < 0.0001; ns: not significant.

LPS was used to induce EC injury to mimic endothelial cell injury during sepsis. To assess the proliferation capacity of ECs, an EdU assay was performed. As shown in Figure 2A,B, the proliferation capacity was decreased after LPS stimulation compared with the control group, while the Wnt5a-overexpressing BMCS coculture increased protective effects. The scratch assay was performed to detect the migration of ECs. The results indicated that LPS stimulation decreased the migration ability of ECs, whereas coculture with different groups of BMSCs partially restored EC migration, with the most pronounced effect observed in the BMSC^Wnt5a^ group (Figure 2C,D). To further assess the angiogenic ability of ECs in vitro, tube formation assays were conducted. As can be seen in Figure 2E,F, ECs in the LPS group exhibited almost no tube formation. However, coculture with different groups of BMSCs promoted tube formation capacity, markedly in the BMSC^Wnt5a^ group. To further clarify the effects of Wnt5a on BMSCs for treatment of LPS-induced EC injury, BMSCs were transfected with the negative control (siNC) or Wnt5a-siRNA. The effects of Wnt5a knockdown in BMSCs on EC recovery were subsequently examined. Compared with the negative control siRNA (siNC-BMSC) group, the proliferation of injured ECs significantly decreased in the Wnt5a-siRNA (siWnt5a-BMSC) group (Figure 3A,B). Consistently, scratch assays revealed that coculture with siWnt5a-BMSC significantly impaired EC migration compared with the siNC-BMSC group (Figure 3C,D). Moreover, the tube formation assay revealed that Wnt5a knockdown in BMSCs markedly attenuated the angiogenic capacity of injured ECs relative to the siNC-BMSC group (Figure 3E,F). Collectively, these results indicated that Wnt5a plays a vital role in the therapeutic effects of BMSCs on LPS-induced EC injury in vitro.

### 3.3. Wnt5a-Overexpressing BMSCs Attenuated Permeability and Increased Expression of ZO-1 and VE-Cadherin on LPS-Injured ECs

**Figure 4:**
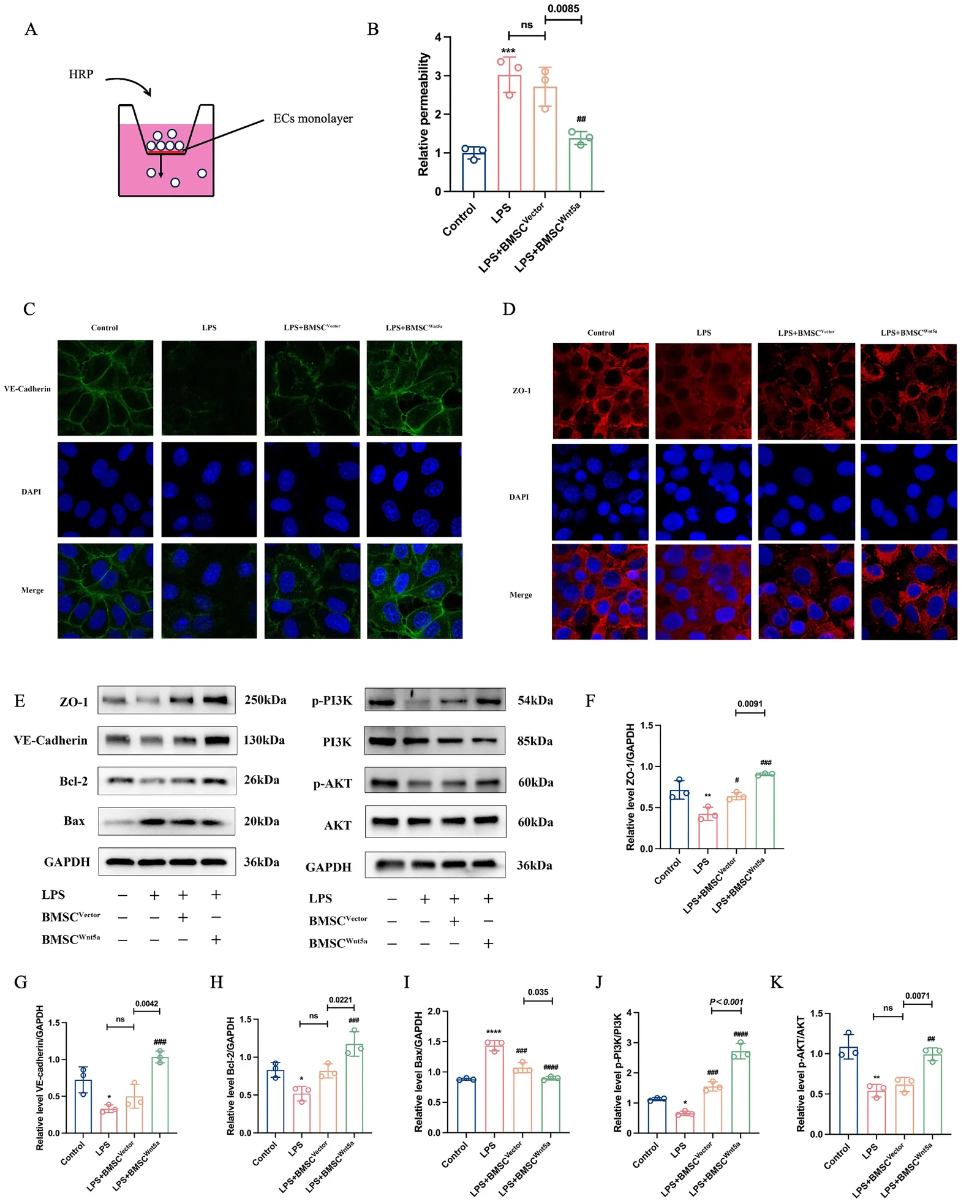
Wnt5a-overexpressing BMSCs attenuated permeability and increased ZO-1 and VE-cadherin expression in ECs, accompanied by enhanced PI3K/AKT signaling. (**A**) Transwell inserts were used to assess the permeability of injured ECs. (**B**) Quantitative analysis of the changes in permeability of EC cocultures with indicated BMSCs. (**C**) Immunofluorescence staining for VE-cadherin (Green) and nuclei (Blue) (400×). (**D**) Immunofluorescence staining for ZO-1 (Red) and nuclei (Blue) (400×). (**E**) Western blot analysis of ZO-1, VE-cadherin, Bcl-2, Bax and PI3K/AKT signaling-related proteins. (**F**–**K**) Densitometric analysis of Western blots. GAPDH served as an internal reference. Data are presented as means ± SEMs, *n* = 3. Compared with the control group, * *p* < 0.05, ** *p* < 0.01, *** *p* < 0.001, **** *p* < 0.0001; compared with the LPS group, # *p* < 0.05, ## *p* < 0.01, ### *p* < 0.001, #### *p* < 0.0001; ns: not significant. The blots were cropped and these uncropped images placed in Supplementary Material: Figures S3–S8.

**Figure 5:**
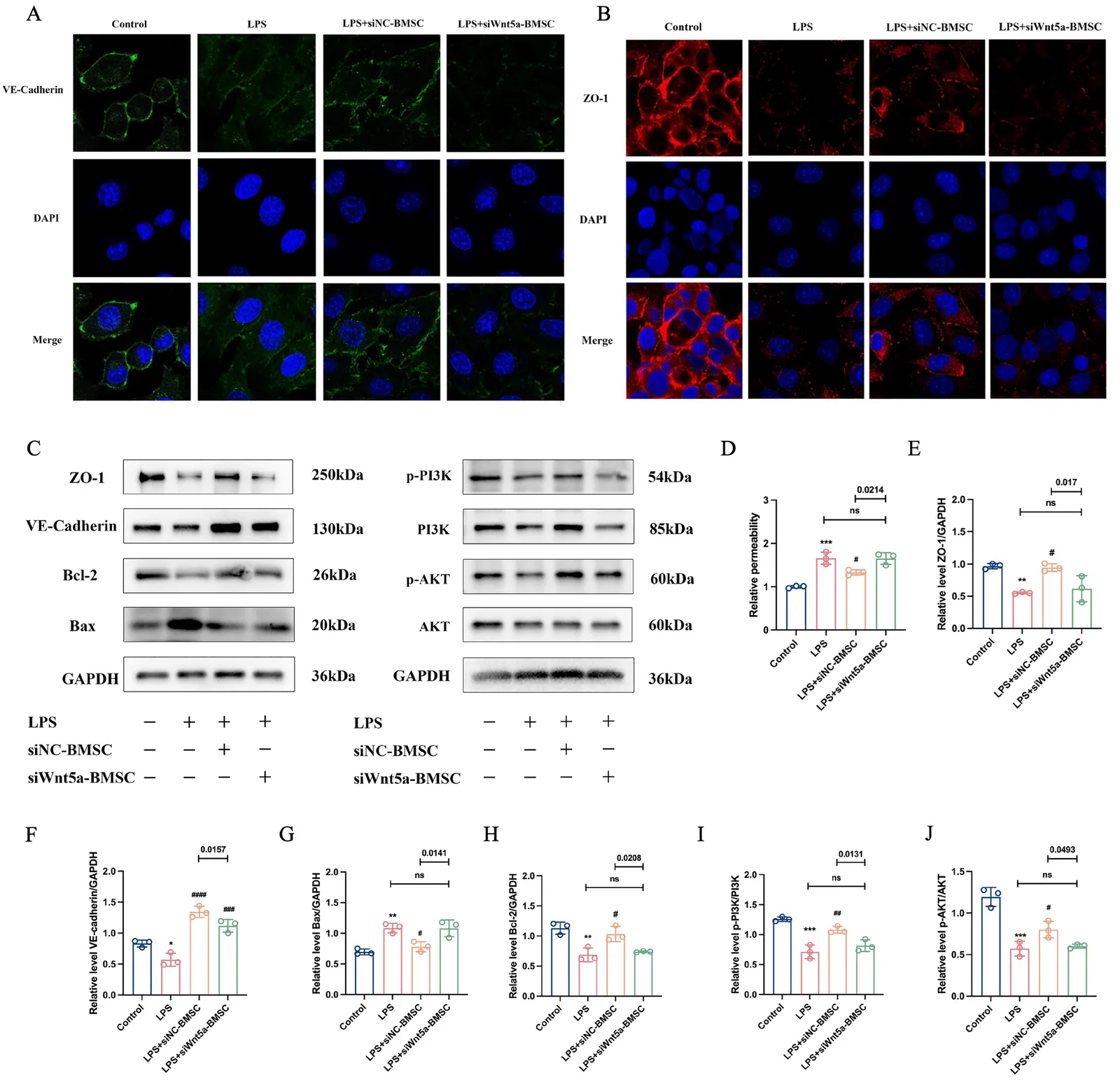
Wnt5a knockdown in BMSCs decreased their protective effects on LPS-induced EC injury. (**A**) Immunofluorescence staining for VE-cadherin (Green) and nuclei (Blue) (400×). (**B**) Immunofluorescence staining for ZO-1 (Red) and nuclei (Blue) (400×). (**C**) Western blot analysis of ZO-1, VE-cadherin, Bcl-2, Bax and PI3K/AKT signaling-related proteins. (**D**–**J**) Densitometric analysis of Western blots. GAPDH served as an internal reference. Data are expressed as means ± SEMs, *n* = 3. Compared with the control group, * *p* < 0.05, ** *p* < 0.01, *** *p* < 0.001; compared with the LPS group, # *p* < 0.05, ## *p* < 0.01, ### *p* < 0.001, #### *p* < 0.0001; ns: not significant. The blots were cropped and these uncropped images placed in Supplementary Material: Figures S9–S14.

To investigate the effects of Wnt5a-overexpressing BMSCs on LPS-induced EC barrier dysfunction, we used transwell inserts to establish the endothelial cell leakage assay (Figure 4A). The results indicated that LPS could significantly increase EC permeability, whereas coculture with BMSCs markedly reduced permeability, with the most pronounced protective effects observed in the BMSC^Wnt5a^ group (Figure 4B). Tight junctions (TJs), adherens junctions (AJs), and gap junctions (GJs) act synergistically to maintain vascular endothelial barrier integrity [22]. Therefore, the expressions of ZO-1 and VE-cadherin were measured by immunofluorescence staining and Western blot analysis. We observed that LPS stimulation significantly decreased the expression of VE-cadherin and ZO-1 in ECs in vitro, while coculture with BMSC^Wnt5a^ markedly restored their expression (Figure 4C,D). Similarly, the Western blot results show that compared with the control group, the protein levels of ZO-1, VE-cadherin, and anti-apoptosis protein Bcl-2 were reduced, while the apoptosis-associated protein Bax was significantly increased in the LPS group. In contrast, the expression of ZO-1, VE-cadherin, and Bcl-2 protein was raised in the BMSC^Wnt5a^ group, in contrast with LPS group and the BMSC^Vector^ group (Figure 4E,K). To further explore the underlying molecular mechanisms of the protective effects of Wnt5a on injured ECs, we assessed the expression of PI3K/AKT signaling. Compared with the LPS group and the BMSC^Vector^ group, the phosphorylation of PI3K and AKT protein was increased in the BMSC^Wnt5a^ group (Figure 4E,K). Intriguingly, while knockdown of Wnt5a on BMSCs decreased the protective effects on LPS-induced EC injury, such as increasing the permeability of ECs and expression of the apoptosis-associated protein Bax, accompanied by reduced expression of ZO-1, VE-cadherin and Bcl-2 (Figure 5A,H). Furthermore, the proteins involved in PI3K/AKT signaling were assessed using Western blotting. The results show that compared with the siNC-BMSC group, the phosphorylation of PI3K and AKT was decreased in the LPS group and the siWnt5a-BMSC group (Figure 5I,J). Taken together, these findings indicated that Wnt5a-overexpressing BMSCs alleviated LPS-induced EC injury, and these protective effects were associated with enhanced PI3K/AKT signaling.

### 3.4. Wnt5a-Overexpressing BMSCs Alleviated LPS-Induced Lung Injury

**Figure 6:**
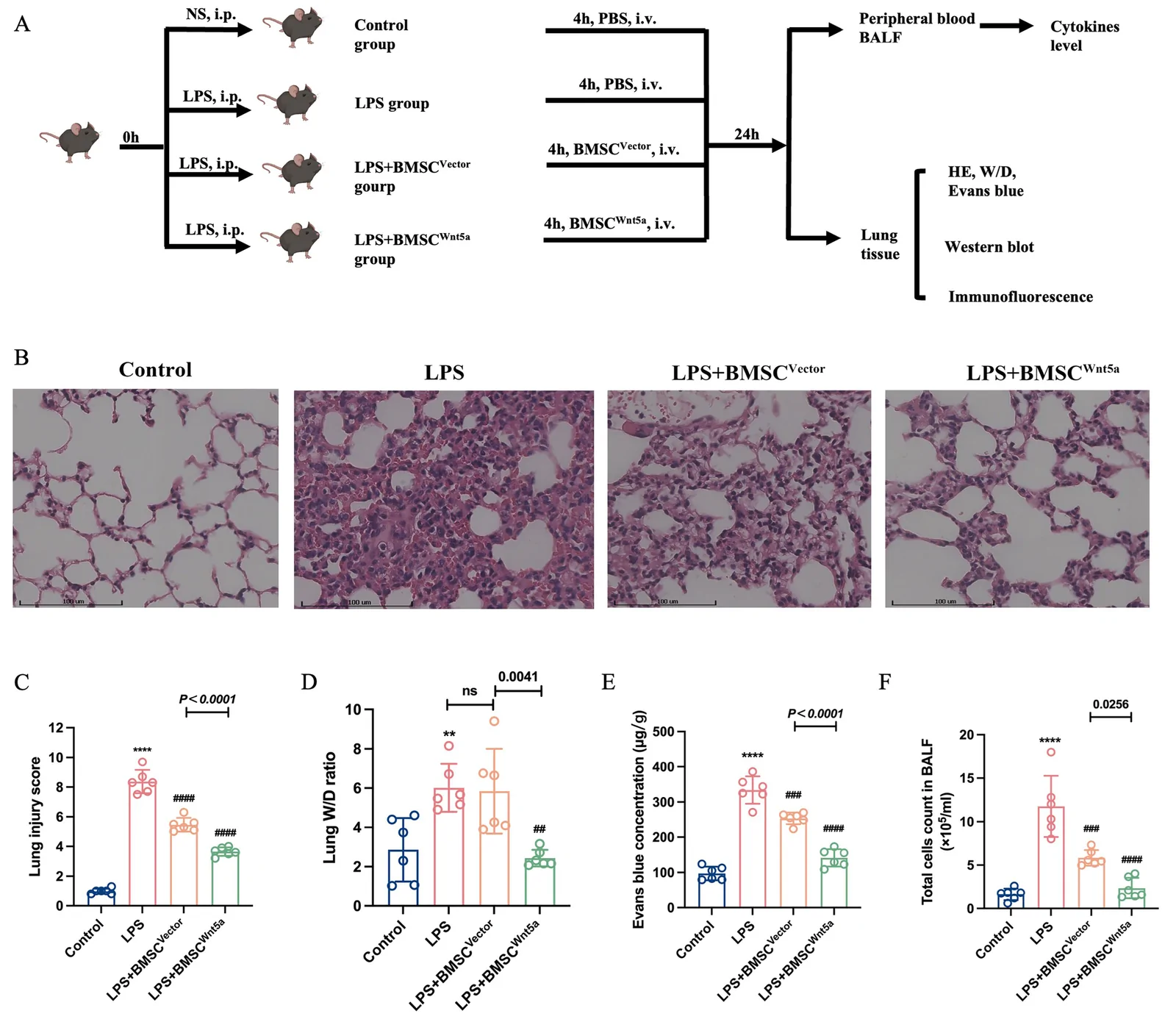
Wnt5a-overexpressing BMSCs attenuated LPS-induced ALI in mice. (**A**) An overview diagram of the experimental protocol. At 4 h after intraperitoneal LPS challenge, mice in the treatment groups received BMSC^Vector^ or BMSC^Wnt5a^ cells suspended in 150 μL of PBS via tail-vein injection, whereas mice in the control and the LPS group received an equivalent volume of PBS. (**B**) The histopathology of mice in each group’s staining with hematoxylin–eosin 24 h after LPS or PBS intraperitoneal injection (magnification: 200×). (**C**) Quantitative analysis shows that BMSC^Wnt5a^ dramatically reduced lung injury scores compared with the LPS group and the BMSC^Vector^ group. (**D**) Lung wet/dry assay in each group. (**E**) Evans blue extravasation assay was used to evaluate pulmonary barrier permeability. (**F**) The detection of total cells counts in BALF. Data are presented as means ± SEMs, *n* = 6. Compared with the control group, ** *p* < 0.01, **** *p* < 0.0001; compared with the LPS group, ## *p* < 0.01, ### *p* < 0.001, #### *p* < 0.0001; ns: not significant.

LPS intraperitoneal injection was used to establish the model of ALI in mice as follows (Figure 6A). Lung tissues from the LPS group showed interstitial thickening, narrowing or obliteration of alveolar spaces and extensive inflammatory cell infiltration in the alveolar walls and interstitium, resulting in significantly higher lung injury scores compared with the control group. In contrast, lung injury was alleviated to varying degrees following BMSC transplantation, with the most pronounced improvement observed in the BMSC^Wnt5a^ group (Figure 6B,C). The dysfunction of pulmonary vascular endothelial cells is one of the typical pathophysiological features of ALI. To evaluate the effects of Wnt5a-overexpressing BMSCs on pulmonary endothelial barrier function, lung wet/dry weight, endothelial barrier extravasation (assessed using Evans Blue dye), and total cell counts were measured. The results showed that LPS administration significantly increased the permeability of the endothelial barrier, as evidenced by elevated Evans blue concentration and increased total cell count in BALF. After transplantation of BMSC^Wnt5a^, the concentration of Evans blue and total cell count remarkably reduced compared with the LPS group and the BMSC^Vector^ group. Notably, no significant difference in W/D weight ratio was observed between the LPS group and the BMSC^Vector^ group (Figure 6D,F). Collectively, these findings indicated that overexpression of Wnt5a could enhance the protective effects of BMSCs on ALI mice by alleviating lung injury and improving pulmonary endothelial barrier dysfunction.

### 3.5. Wnt5a-Overexpressing BMSCs Reduced the Concentrations of Inflammatory Cytokines in LPS-Induced ALI

**Figure 7:**
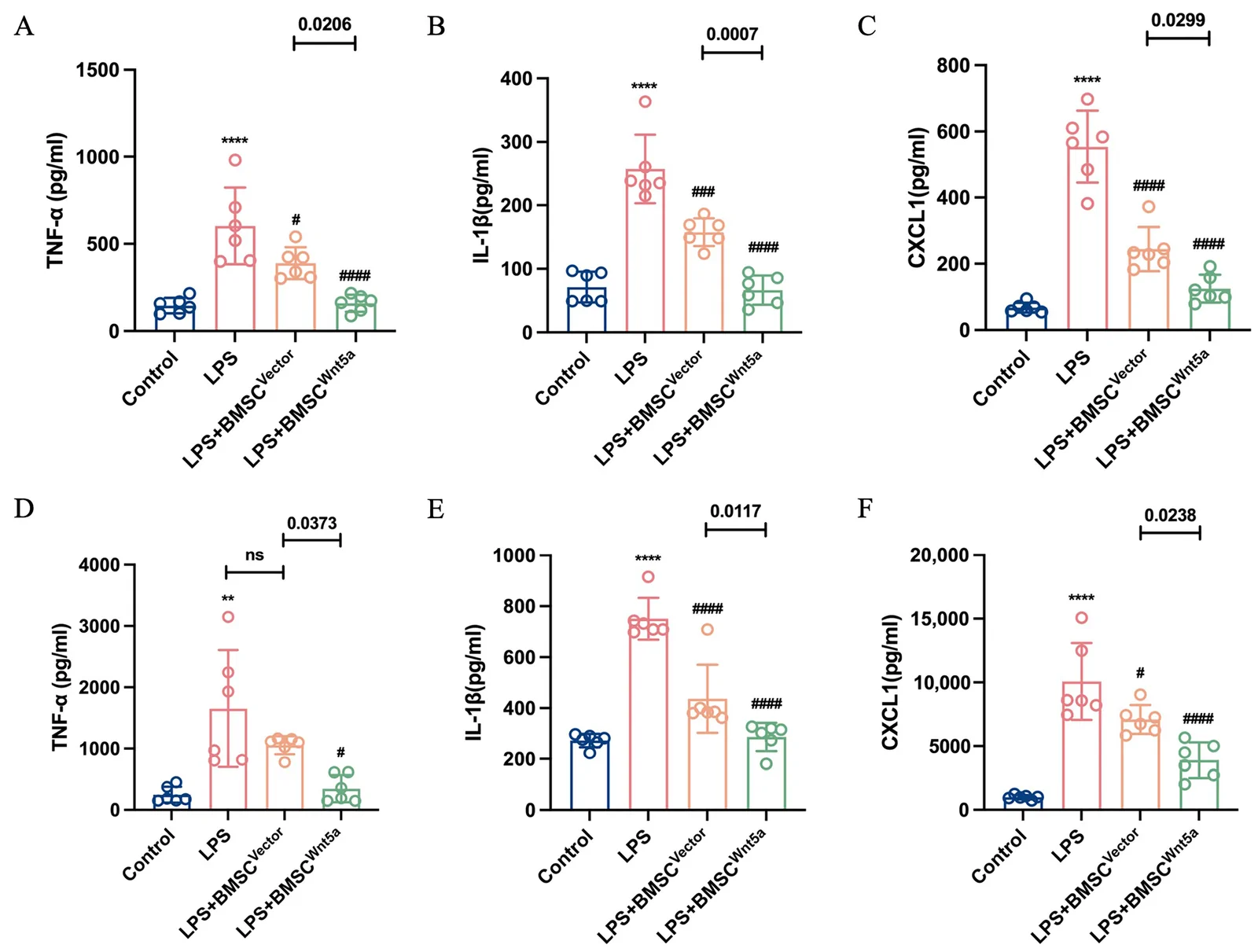
Wnt5a-overexpressing BMSCs reduced inflammatory cytokine concentrations in LPS-induced ALI. (**A**–**C**) Concentrations of TNF-α, IL-1β, and CXCL1 in BALF. (**D**–**F**) Concentrations of TNF-α, IL-1β, and CXCL1 in serum. Data are presented as means ± SEMs, *n* = 6. Compared with the control group, ** *p* < 0.01, **** *p* < 0.0001; compared with the LPS group, # *p* < 0.05, ### *p* < 0.001, #### *p* < 0.0001; ns: not significant.

Early elevations of proinflammatory cytokine concentrations during ALI indicate an excessive inflammatory response. We therefore measured the concentrations of inflammatory cytokines in mice using ELISA kits. The results showed that the concentrations of TNF-α, IL-1β, and CXCL1 in BALF and serum were raised in the LPS group compared with the control group. The BMSC^Wnt5a^ group exhibited lower concentrations of inflammatory cytokines than the LPS group and the BMSC^Vector^ group (Figure 7A–F). These findings suggested that overexpression of Wnt5a enhanced the protective effects of BMSCs to modulate the inflammatory response in ALI mice.

### 3.6. Wnt5a-Overexpressing BMSCs Exerted Protective Effects Associated with Increased ZO-1 and VE-Cadherin Expression and Enhanced PI3K/AKT Signaling

**Figure 8:**
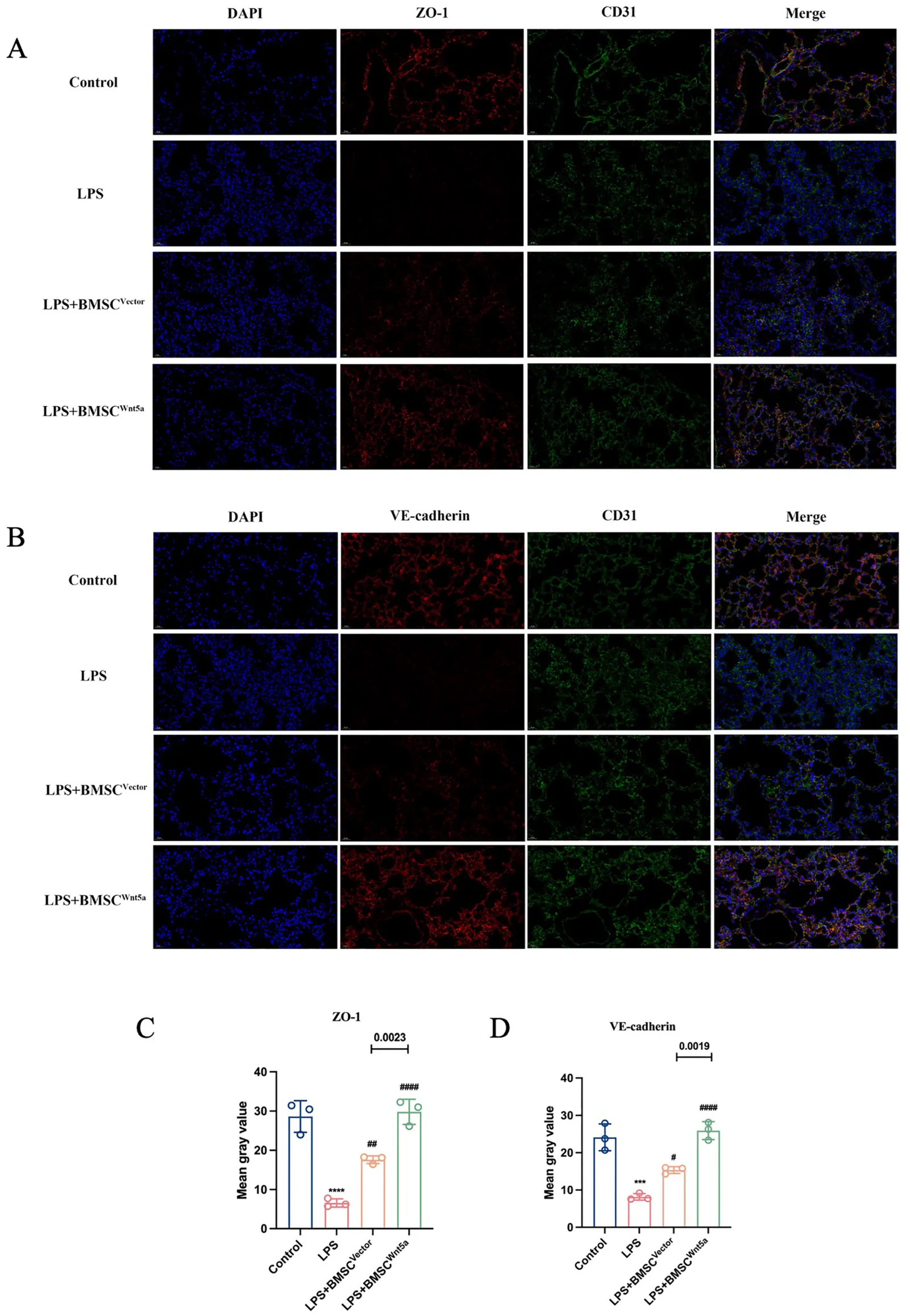
Wnt5a-overexpressing BMSCs increased ZO-1 and VE-cadherin expression in lung tissues of LPS-induced ALI mice. (**A**) Immunofluorescence staining for ZO-1 (green) and nuclei (blue) (400×). (**B**) Immunofluorescence staining for VE-cadherin (red) and nuclei (blue) (400×). (**C**) Quantitative analysis of ZO-1 expression. (**D**) Quantitative analysis of VE-cadherin. Data are presented as means ± SEMs, *n* = 3. Compared with the control group, *** *p* < 0.001, **** *p* < 0.0001; compared with the LPS group, # *p* < 0.05, ## *p* < 0.01, #### *p* < 0.0001.

**Figure 9:**
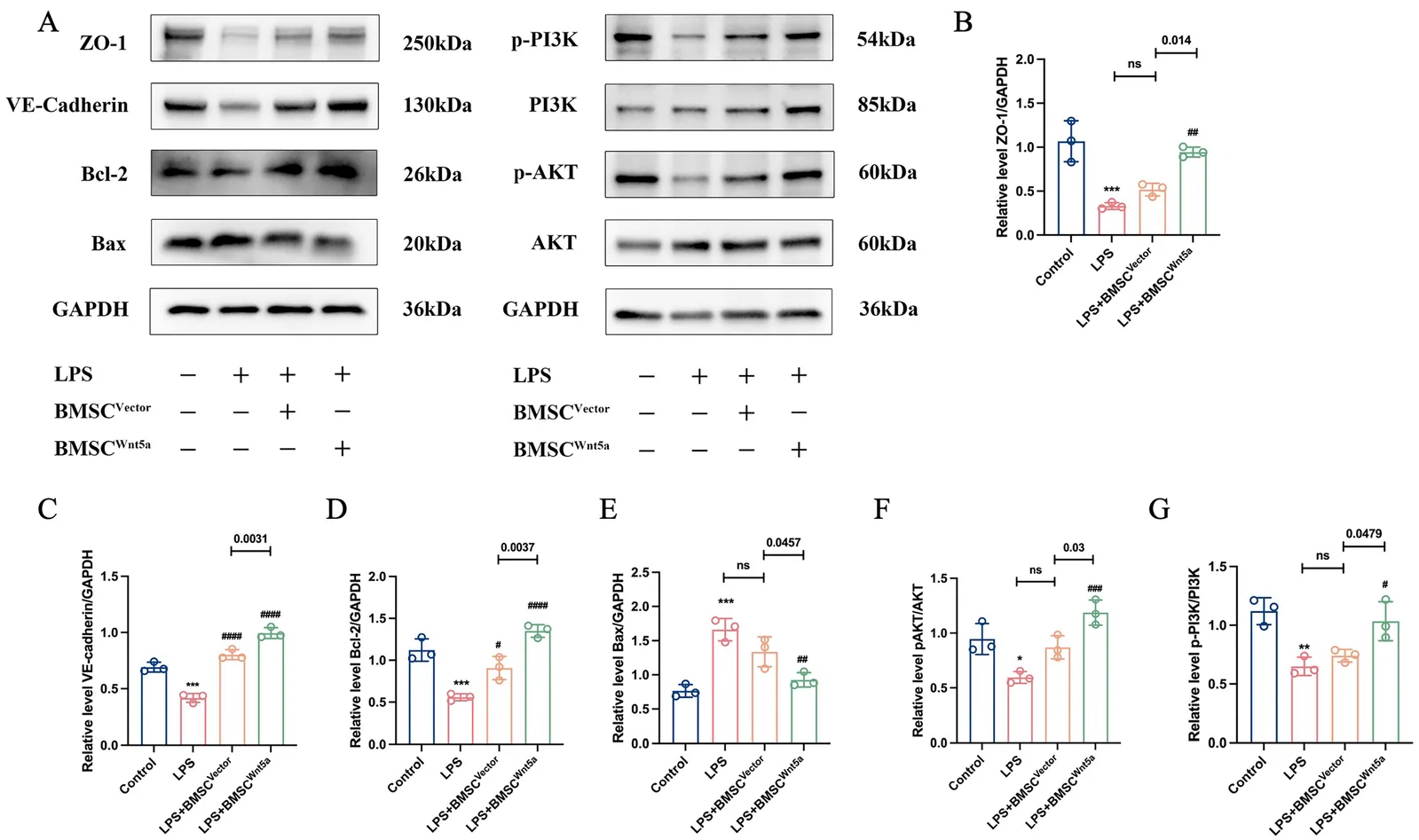
Wnt5a-overexpressing BMSC treatment was associated with preservation of endothelial junctional proteins, an anti-apoptotic protein profile and enhanced PI3K/AKT signaling in LPS-induced ALI. (**A**) Western blot analysis of ZO-1, VE-cadherin, Bcl-2, Bax and PI3K/AKT signaling-related proteins in lung tissues. (**B**–**G**) Densitometric analysis of Western blots. GAPDH served as an internal reference. Data are presented as means ± SEMs, *n* = 3. Compared with the control group, * *p* < 0.05, ** *p* < 0.01, *** *p* < 0.001; compared with the LPS group, # *p* < 0.05, ## *p* < 0.01, ### *p* < 0.001, #### *p* < 0.0001; ns: not significant. The blots were cropped and these uncropped images placed in Supplementary Material: Figures S15–S20.

To explore whether Wnt5a-overexpressing BMSCs exerted protective effects by upregulating the expression of ZO-1 and VE-cadherin in lung tissues in ALI mice, immunofluorescence staining and Western blot analyses were performed. The immunofluorescence of lung tissues showed that compared with the control group, the expressions ZO-1 and VE-cadherin were dramatically reduced in the LPS group. In contrast, transplantation of BMSCs increased expression of ZO-1 and VE-cadherin, with the most prominent restoration observed in the BMSC^Wnt5a^ group (Figure 8A–D). Consistent with these findings, Western blots of lung tissues also indicated that the expressions of ZO-1 and VE-cadherin were decreased after LPS administration. The BMSC^Wnt5a^ treatment increased ZO-1 and VE-cadherin protein expression compared with the BMSC^Vector^ group (Figure 9A–C). Endothelial cell apoptosis contributes to barrier dysfunction, increased vascular permeability, and exacerbation of lung injury [23]. To further evaluate the effects of Wnt5a-overexpressing BMSCs on apoptosis in ALI mice, the expression of apoptosis-associated proteins (Bax, Bcl-2) in lung tissues was assessed by Western blotting. The expression of the apoptosis-associated protein Bax was significantly decreased and that of anti-apoptosis protein was dramatically increased in the BMSC^Wnt5a^ group compared with the LPS group and the BMSC^Vector^ group (Figure 9A,D–G). Taken together, these results suggested that the protective effects of Wnt5a-overexpressing BMSCs against LPS-induced ALI were associated with increased endothelial junction protein expression, altered apoptosis-related protein expression, and enhanced PI3K/AKT signaling.

## 4. Discussion

In this study, we demonstrated that Wnt5a-overexpressing BMSCs enhanced EC proliferation, migration, tube formation and barrier integrity and shifted apoptosis-related protein expression towards an anti-apoptotic profile following LPS-induced injury in vitro. Furthermore, we established the ALI model by LPS and transplanted Wnt5a-overexpressing BMSCs into mice via tail-vein injection. We observed that overexpression of Wnt5a optimized the therapeutic effects of BMSCs on ALI, as indicated by reduced pulmonary edema, lung injury, and concentrations of inflammatory cytokines; a shift in apoptosis-related protein expression towards an anti-apoptotic profile; and restoration of ZO-1 and VE-cadherin expression in lung tissues. These protective effects were accompanied by enhanced PI3K/AKT signaling.

Despite extensive preclinical evidence for MSC-based therapies, poor post-transplantation survival and limited engraftment remain barriers to clinical translation. Genetic modification may improve MSC retention or augment selected therapeutic functions. For instance, lentivirus-mediated overexpression of CXC chemokine receptor 7 (CXCR7) in rat BMSCs significantly improved outcomes in phosgene-induced ALI [24], whereas overexpression of the angiotensin II type 2 receptor (AT2R) enhanced BMSC retention, reduced pulmonary vascular permeability and attenuated inflammation in LPS-induced ALI [25]. Consistent with these findings, our previous study demonstrated that ghrelin pretreatment significantly enhanced the therapeutic efficacy of BMSCs against LPS-induced EC injury. Transcriptomic analysis of BMSC-conditioned medium revealed a marked upregulation of Wnt5a across treatment groups, suggesting that elevated Wnt5a expression may be a key contributor to BMSC-mediated improvement of endothelial function [19].

In this study, we overexpressed Wnt5a in BMSCs by lentivirus transduction and cocultured them with ECs to evaluate the therapeutic effects on LPS-induced endothelial injury. Our results demonstrated that Wnt5a overexpression enhanced the ability of BMSCs to promote endothelial proliferation, migration and tube formation and to reduce endothelial permeability under LPS challenge. Conversely, Wnt5a knockdown weakened these beneficial effects. These findings suggest that Wnt5a may be involved in BMSC-mediated endothelial repair and may contribute to enhanced paracrine protection. However, Wnt5a should not be regarded as a purely pro-inflammatory molecule because its biological effects are strongly influenced by cellular source, receptor repertoire, disease stage, and local microenvironment. Liu et al. reported that exogenous Wnt5a promoted alveolar type II (ATII) cell differentiation from MSCs through non-canonical Wnt signaling, thereby enhancing alveolar epithelial repair [26]. Similarly, Xu et al. reported that hyperoxia, which is commonly used in neonatal ARDS, downregulated Wnt5a expression both in vivo and in vitro and inhibited ATII-to-ATI transdifferentiation, a process crucial for alveolar epithelial barrier integrity [27]. In contrast, recent studies have reported that Wnt5a can be induced by LPS/IFN-γ and may promote inflammatory cytokine release through FZD5/CaMKII-, JNK-, and NF-κB-related signaling pathways, thereby contributing to sepsis-associated inflammatory tissue injury [28,29,30]. Nevertheless, Bergenfelz et al. reported that Wnt5a can induce a tolerogenic macrophage phenotype, increase anti-inflammatory IL-10 production, and suppress classical TLR4/NF-κB signaling, suggesting a potential role in immune modulation or inflammation resolution under specific conditions [31]. In the present study, Wnt5a was not administered systemically as a free recombinant protein; instead, it was overexpressed in BMSCs and delivered as part of an MSC-based cellular product. Given that MSCs exert their therapeutic effects primarily through paracrine and immunomodulatory mechanisms, the observed reductions in TNF-α, IL-1β, and CXCL1 may not reflect a direct anti-inflammatory effect of Wnt5a alone. Rather, they may represent the integrated paracrine and immunomodulatory effects of Wnt5a-modified BMSCs within the local inflammatory microenvironment.

The endothelial cell barrier is maintained in part by TJs, AJs, and GJs. ZO-1, a key scaffolding protein of TJs, bridges transmembrane adhesion proteins and the cytoskeleton, thereby restricting the paracellular passage of solutes [32]. VE-cadherin, a major component of AJs, mediates intercellular adhesion through its extracellular domain, while its intracellular domain interacts with β-catenin or α-catenin to anchor the complex to the actin cytoskeleton, thereby strengthening cell-to-cell adhesion. Disruption of these junctional proteins is a central mechanism underlying endothelial barrier dysfunction in ALI/ARDS. Immunofluorescence and Western blot analyses revealed that LPS stimulation markedly reduced the expression of the endothelial junction proteins VE-cadherin and ZO-1. Coculture with BMSCs from different groups partially restored the expression of these proteins, with the most pronounced effect observed in the BMSC^Wnt5a^ group. In contrast, knockdown of Wnt5a significantly weakened the ability of BMSCs to rescue VE-cadherin and ZO-1 expression in injured ECs.

Endothelial cell apoptosis contributes to endothelial barrier disruption and increased pulmonary vascular permeability in ALI/ARDS. Recent studies have shown that severe infection, LPS, and other injurious stimuli can induce endothelial apoptosis, thereby compromising pulmonary vascular barrier integrity, promoting pulmonary edema, and amplifying inflammatory responses [24,25]. In the present study, LPS challenge increased the expression of the pro-apoptotic protein Bax and decreased the expression of the anti-apoptotic protein Bcl-2 in ECs and lung tissues from ALI mice. Compared with control BMSCs, Wnt5a-overexpressing BMSCs reduced Bax expression and increased Bcl-2 expression, whereas Wnt5a knockdown attenuated these changes. These findings suggest that Wnt5a modification enhances the ability of BMSCs to modulate apoptosis-related protein expression in LPS-injured endothelial cells.

Cytokine storm is an immunopathological process triggered by the excessive release of pro-inflammatory cytokines, such as TNF-α, ILs, and IFNs. Under physiological conditions, cytokines released by immune cells contribute to host defense against invading pathogens. However, uncontrolled immune activation and excessive cytokine release can trigger a cytokine storm, leading to widespread tissue injury [33]. During ALI/ARDS, damage to alveolar epithelial cells and pulmonary capillary endothelial cells impairs alveolar fluid clearance, facilitating pathogen recognition by pattern recognition receptors on macrophages and subsequent activation of resident pulmonary macrophages. Intracellular signaling cascades then stimulate the production and release of pro-inflammatory cytokines and chemokines, including TNF-α, IL-1β, IL-6, and CXCL1, which promote the recruitment and activation of neutrophils and monocytes. Activated neutrophils further amplify inflammatory responses, ultimately exacerbating lung injury, pulmonary edema, and hyaline membrane formation [2]. In our in vivo experiments, an ALI model was established by intraperitoneal injection of LPS. The total cell count in BALF was determined, and ELISA was performed to measure the concentrations of TNF-α, IL-1β, and CXCL1 in both serum and BALF. We observed that LPS administration significantly increased total BALF cell counts and elevated the concentrations of TNF-α, IL-1β, and CXCL1. Treatment with BMSCs in different groups markedly reduced the inflammatory cytokine concentrations in ALI mice, with Wnt5a-overexpressing BMSCs exhibiting the most pronounced anti-inflammatory effects. These results indicated that Wnt5a overexpression enhances the ability of BMSCs to alleviate pulmonary inflammation in ALI mice.

The PI3K/AKT signaling pathway regulates endothelial cell proliferation, survival, and angiogenic function and contributes to the maintenance of vascular barrier integrity [34]. Its involvement in endothelial protection and lung injury has been demonstrated in several studies. Simvastatin promoted the repair of LPS-induced endothelial injury by activating the PI3K/AKT pathway [35], whereas phosphatidylethanolamine-binding protein 4 (PEBP4) deficiency further suppressed PI3K/AKT signaling and aggravated pulmonary edema and impaired alveolar fluid clearance in LPS-induced ALI [36]. In addition, Mizuta et al. demonstrated that exosomes derived from adipose tissue mesenchymal stem cells alleviated histone-induced endothelial injury and lung damage, with these effects involving PI3K/AKT activation [37]. Consistent with these observations, the protective effects of Wnt5a-overexpressing BMSCs in the present study were accompanied by increased PI3K and AKT phosphorylation in endothelial cells and lung tissues, whereas Wnt5a knockdown reduced their phosphorylation. These findings indicate a positive association between Wnt5a expression in BMSCs and enhanced PI3K/AKT signaling in injured endothelial cells. Nevertheless, future studies incorporating pathway-specific inhibitors may help to further elucidate the potential contribution of PI3K/AKT signaling to the protective effects of Wnt5a-modified BMSCs in ALI/ARDS.

Despite these promising findings, several limitations of this study should be acknowledged. First, only an LPS-induced ALI model was used. Although this model is widely used to investigate endotoxin-mediated acute inflammatory lung injury, it does not fully recapitulate the heterogeneous pathological features of human ALI/ARDS. Therefore, further validation in additional ALI/ARDS models is required. Second, the experimental design involved cross-species systems, which may have introduced species-specific differences in immune responses and paracrine effects. In addition, EA.hy926 cells were used as the in vitro endothelial injury model. Although this cell line is stable and highly reproducible, it cannot fully represent the physiological characteristics of pulmonary microvascular endothelial cells, which may limit the clinical extrapolation of our in vitro findings. Third, the clinical translation of genetically modified BMSCs requires comprehensive long-term safety evaluation and a robust monitoring framework, particularly given that sustained or excessive PI3K/AKT activation may increase the risk of abnormal proliferation, fibrosis, or tumor-related events. Fourth, the impact of Wnt5a overexpression on the broader paracrine secretome of BMSCs remains unclear and should be further explored using comprehensive proteomic and transcriptomic analyses.

## 5. Conclusions

Our findings indicate that overexpression of Wnt5a enhanced the protective effects of BMSCs against LPS-induced ALI by mitigating inflammation and improving endothelial barrier function. These protective effects were accompanied by enhanced PI3K/AKT signaling, providing new insights for developing BMSC-based therapeutic strategies for ALI/ARDS.

## Data Availability

The data that support the findings of the current study are available from the corresponding author on reasonable request.

## Supplementary Materials

The following supporting information can be downloaded at: https://www.mdpi.com/article/10.3390/biom16091356/s1. The original uncropped Western blot images are available in the Supplementary Materials.

## Abbreviations

BMSC	Bone marrow mesenchymal stem cell
ALI	Acute lung injury
ARDS	Acute respiratory distress syndrome
LPS	Lipopolysaccharide
Wnt5a	Wingless-type MMTV integration site family member 5A
ZO-1	Zonula occludens-1
VE-cadherin	Vascular endothelial cadherin
TNF-α	Tumor necrosis factor α
IL-1β	Interleukin 1β
W/D	Wet/dry weight ratio
ELISA	Enzyme-linked immunosorbent assay
