# Supplementary material for: Therapeutic Effects of Wnt5a-Overexpressing Bone Marrow Mesenchymal Stem Cells on Endothelial Barrier Repair in Acute Lung Injury
Source: Biomolecules. 2026 Sep 17;16(9):1356. doi: 10.3390/biom16091356 (PMC13604216; doi:10.3390/biom16091356)

Figure S1. Uncropped gels for Western blots in Figure 1 of Wnt5a overexpression

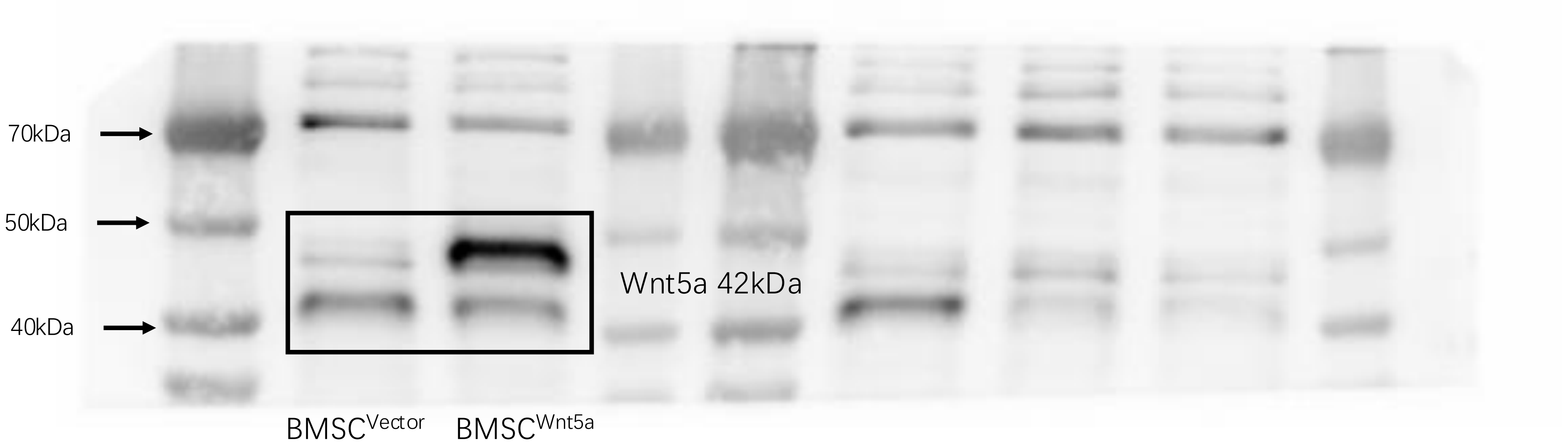

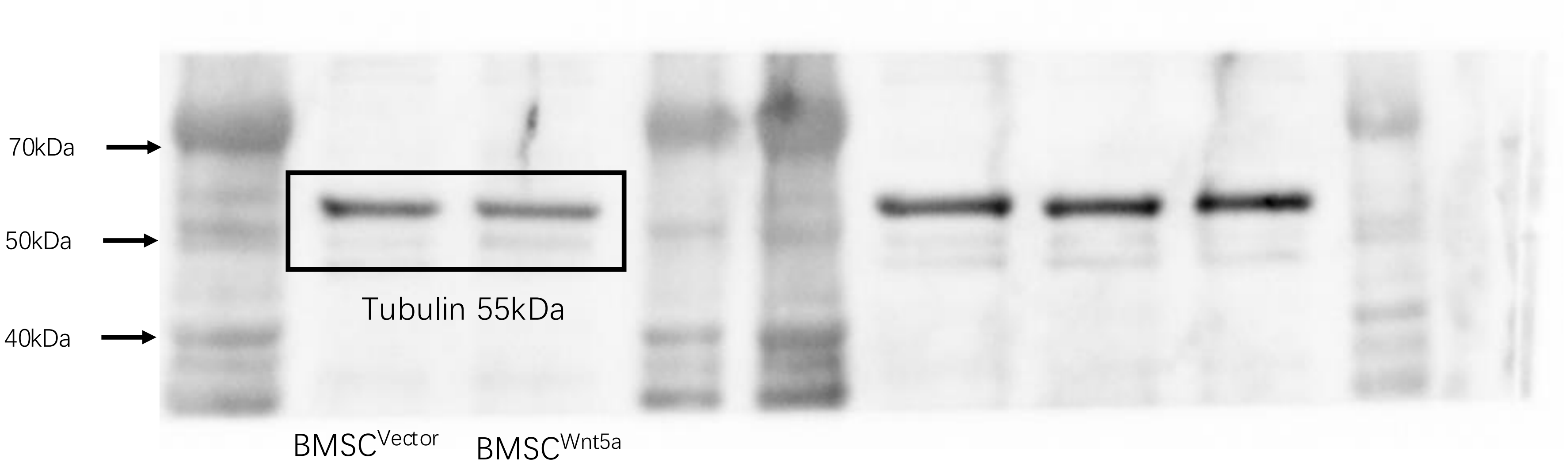

Figure S2 Uncropped gels for Western blots in Figure 1 of Wnt5a knockdown.

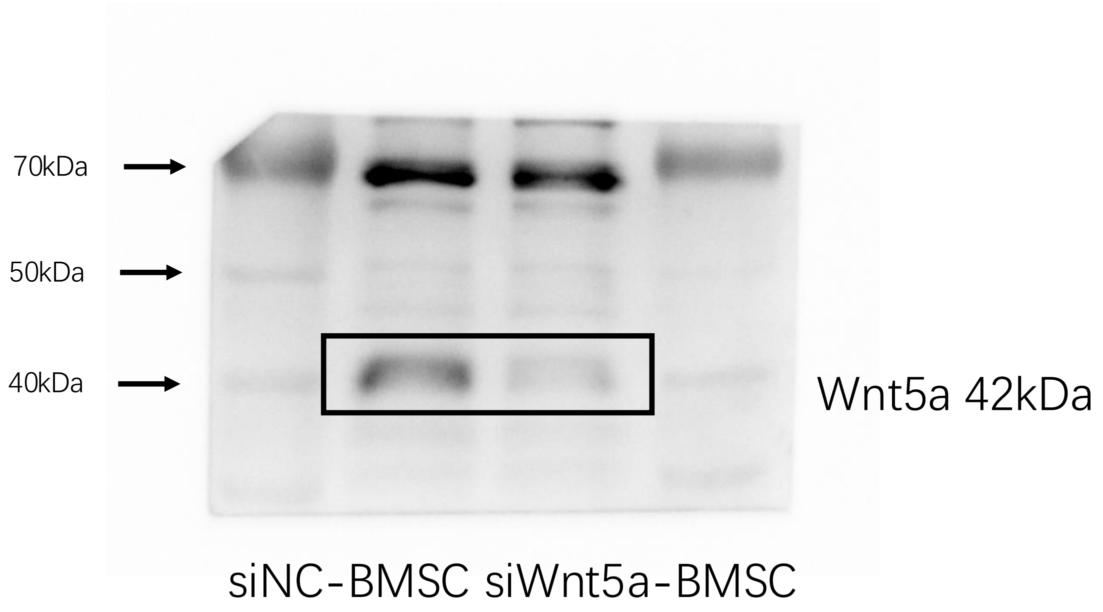

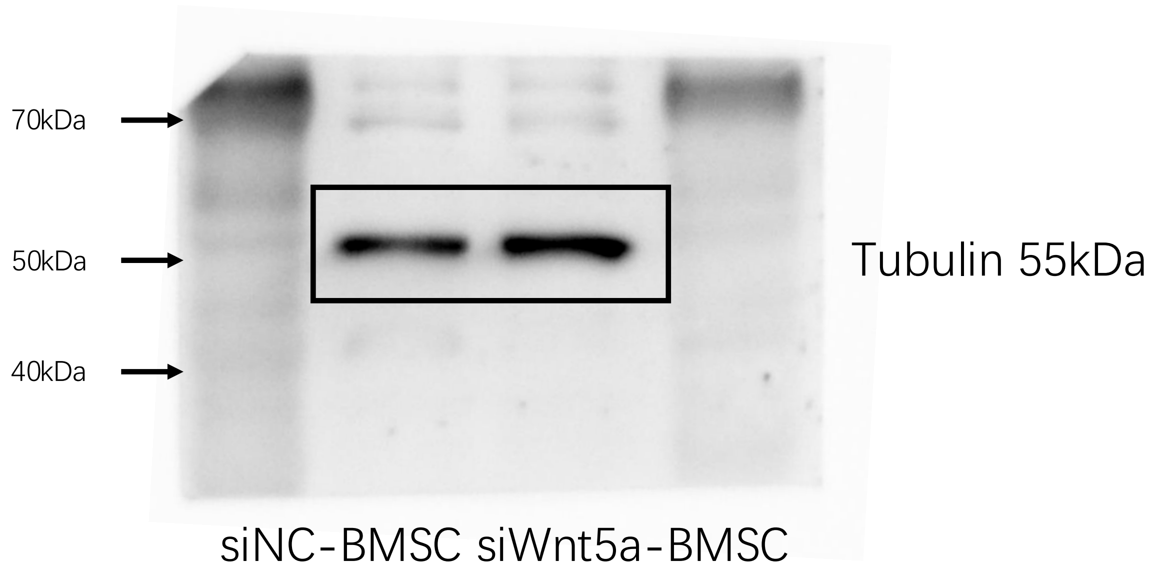

Figure S3. Uncropped and repeated gels for Western blots in Figure 4 of ZO-1

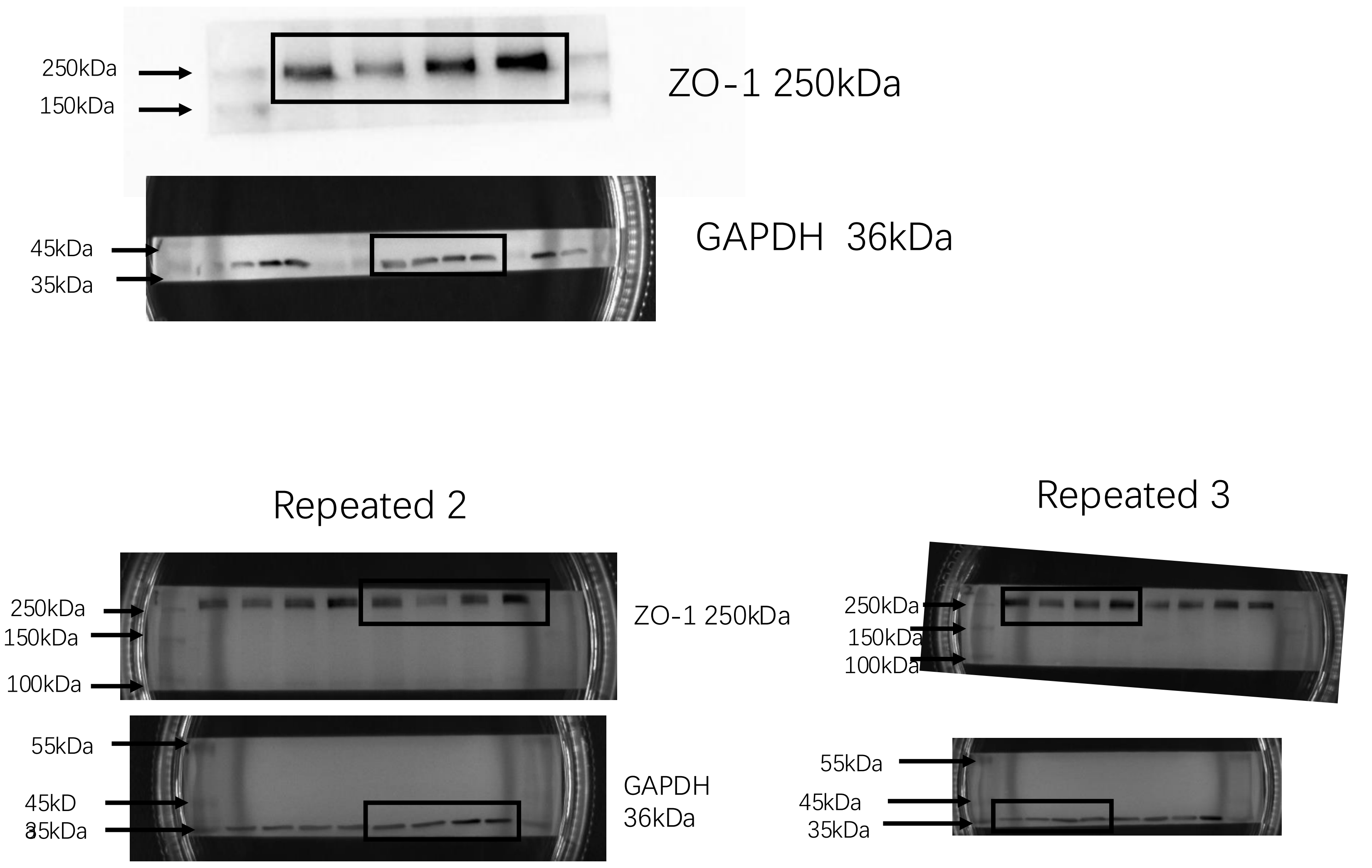

Figure S4. Uncropped and repeated gels for Western blots in Figure 4 of VE-cadherin

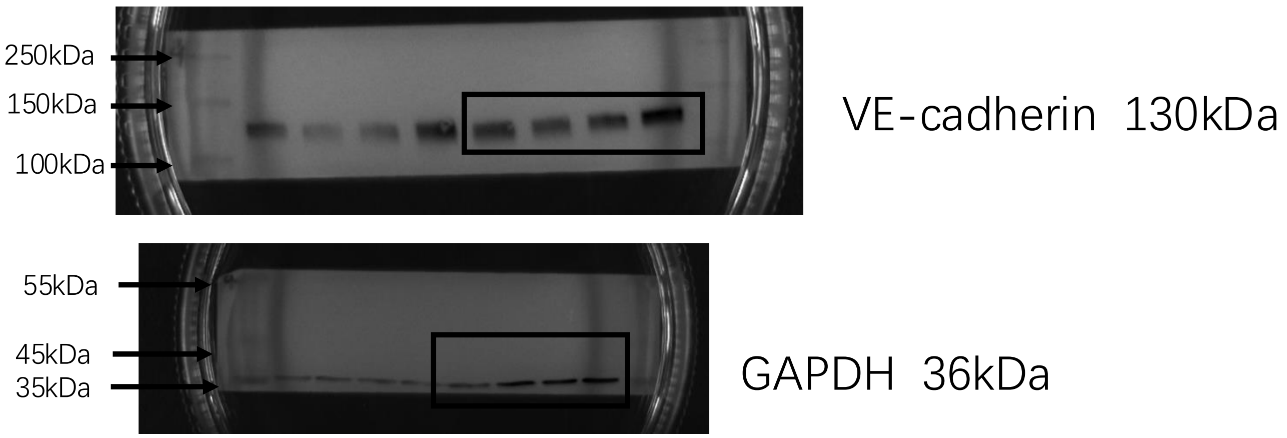

Repeated 2

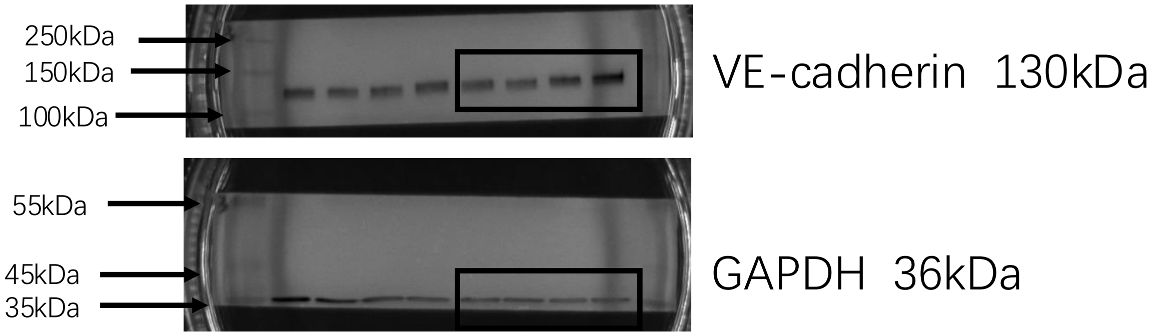

Repeated 3

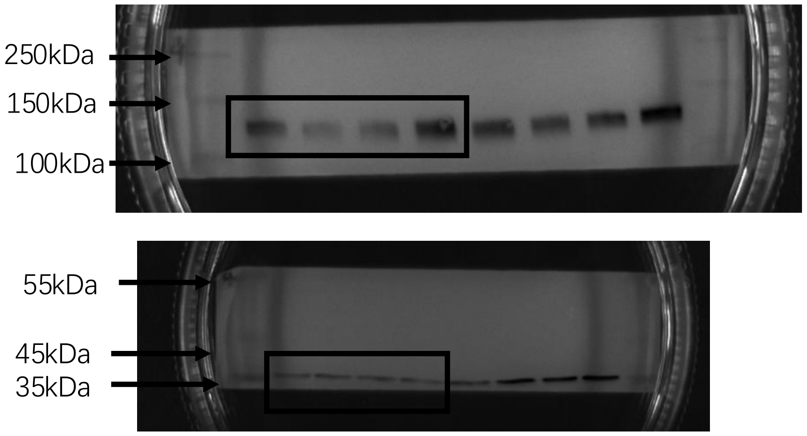

Figure S5. Uncropped and repeated gels for Western blots in Figure 4 of Bcl-2

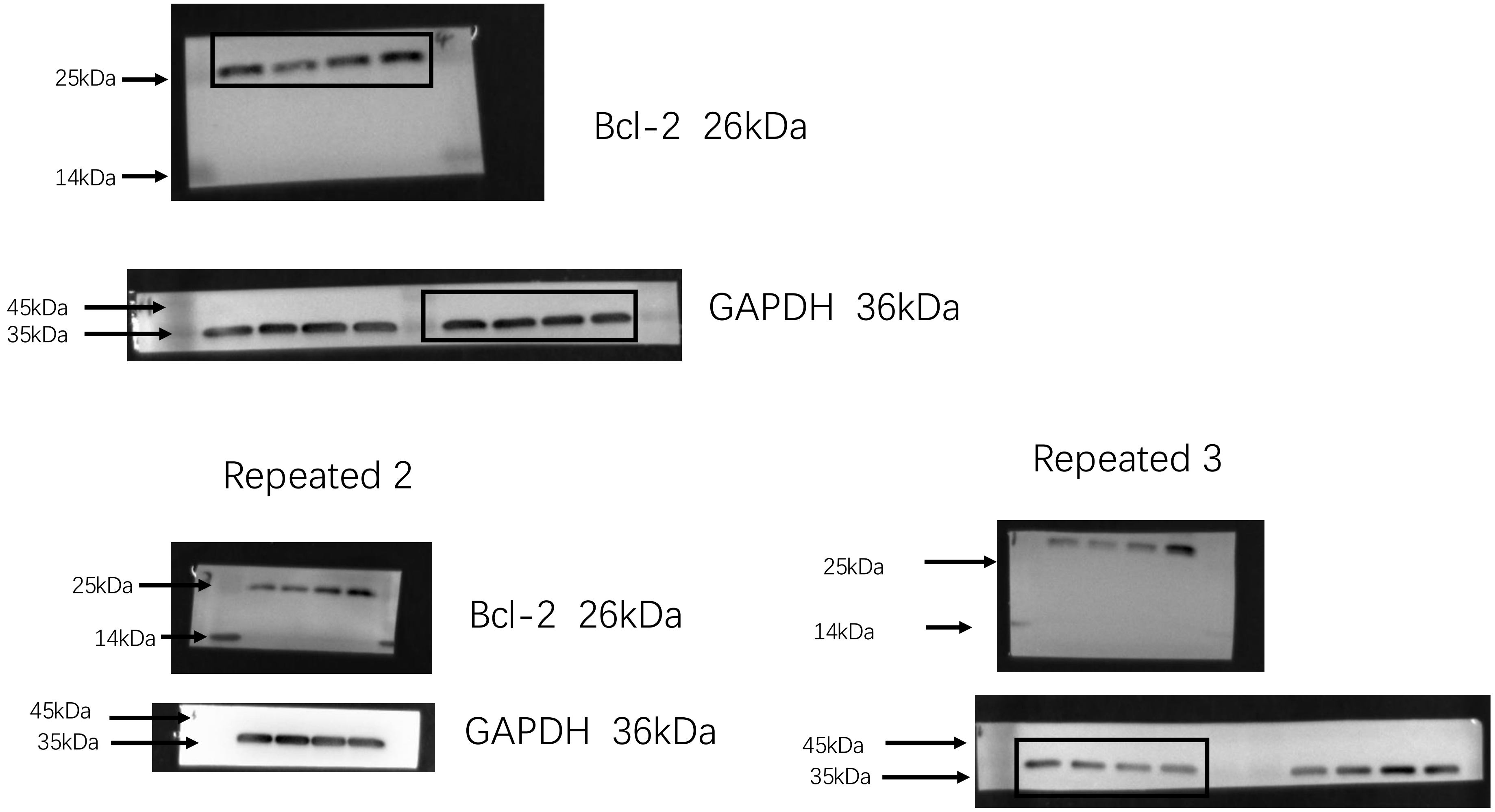

Figure S6. Uncropped and repeated gels for Western blots in Figure 4 of Bax

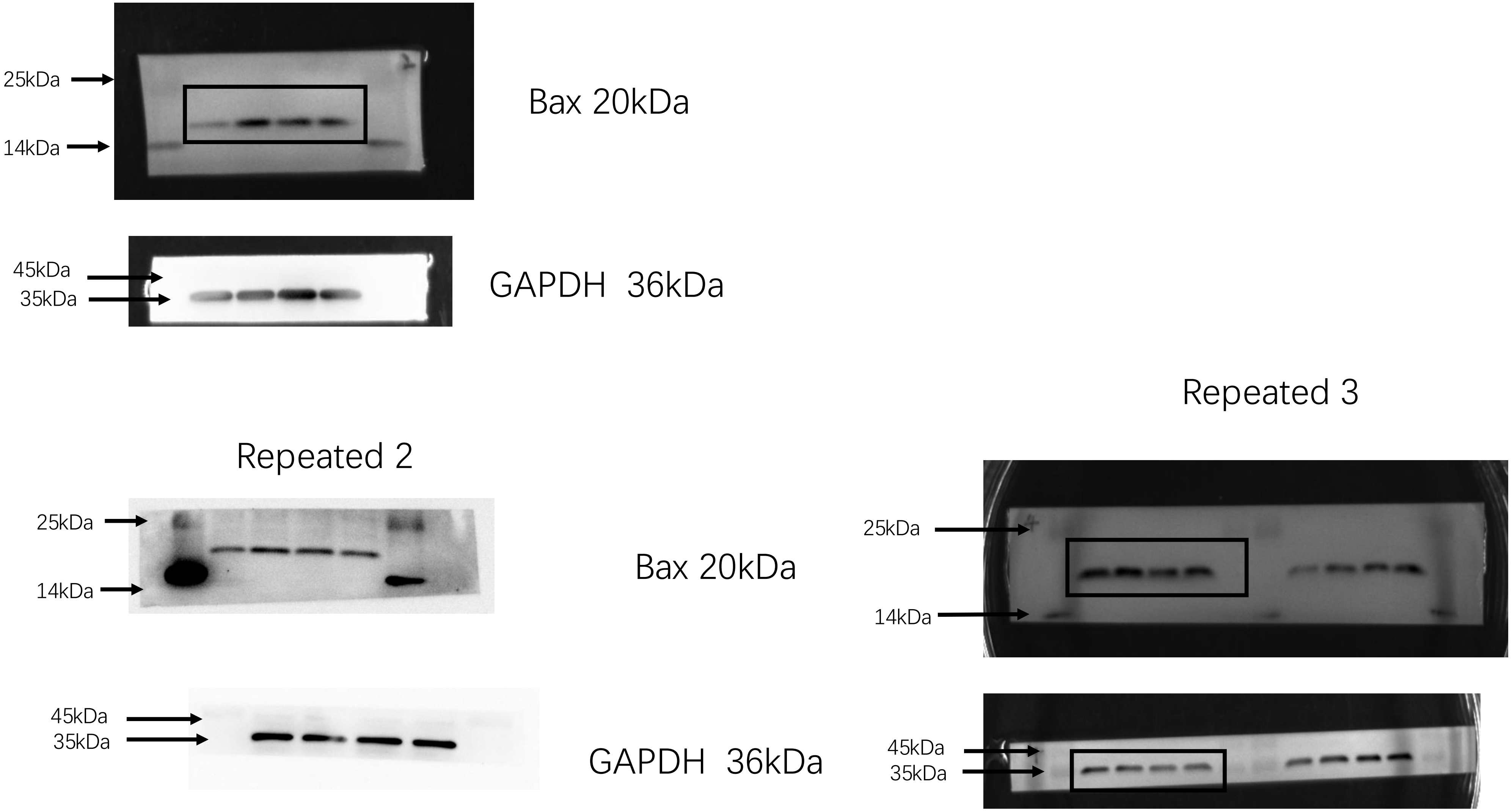

Figure S7. Uncropped and repeated gels for Western blots in Figure 4 of p-PI3K、PI3K

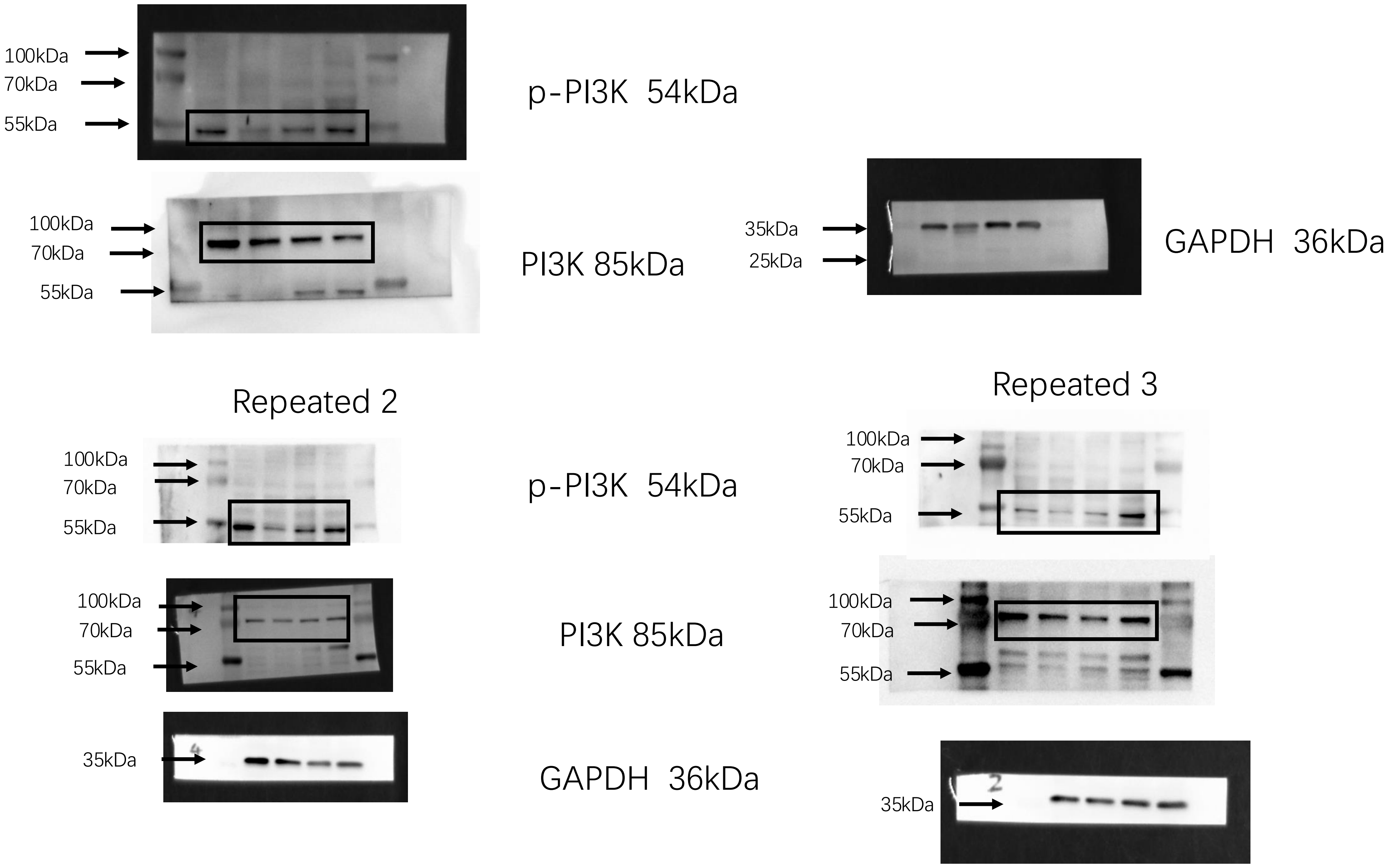

Figure S8. Uncropped and repeated gels for Western blots in Figure 4 of p-AKT、AKT

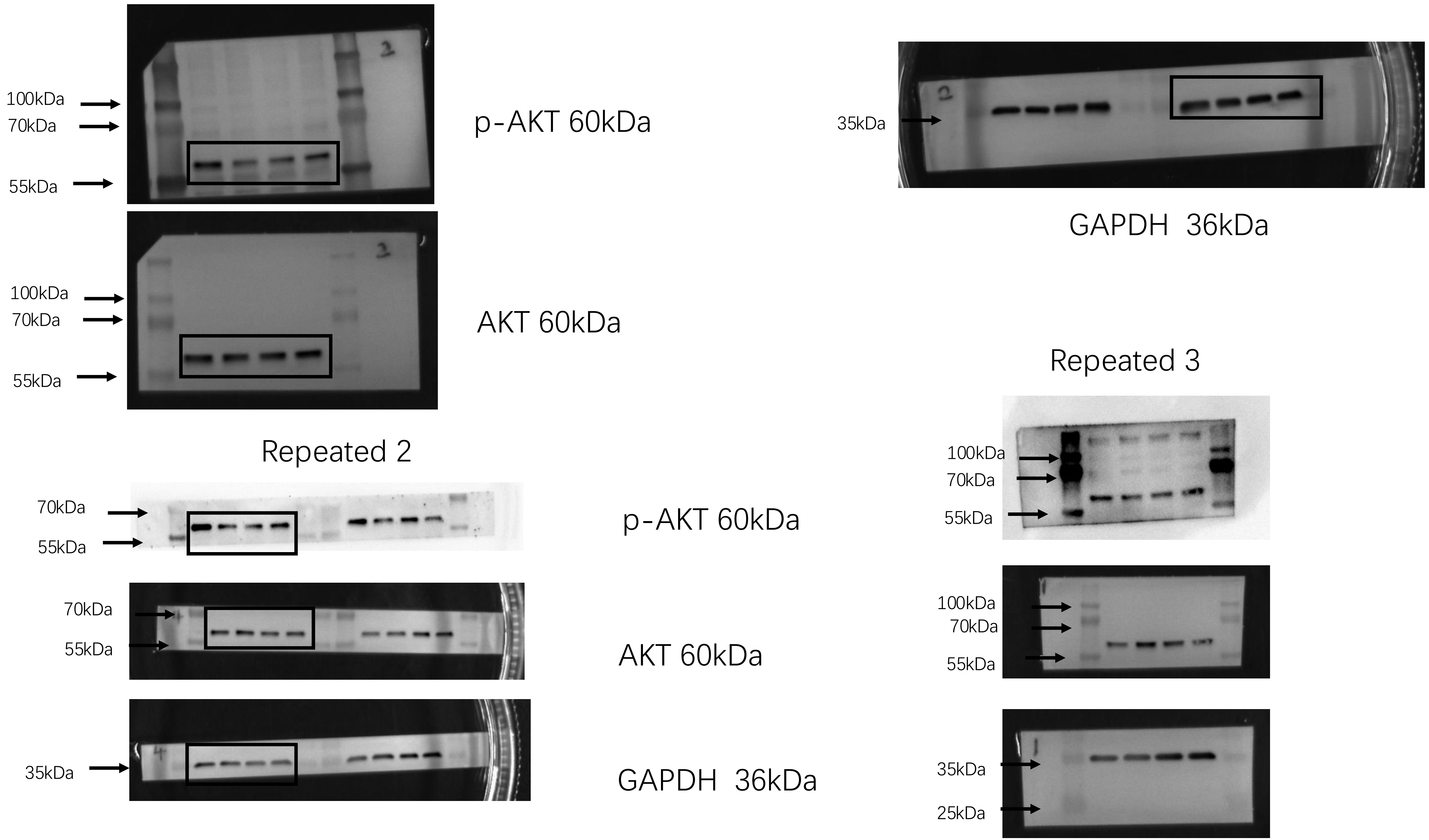

Figure S9. Uncropped and repeated gels for Western blots in Figure 5 of ZO-1

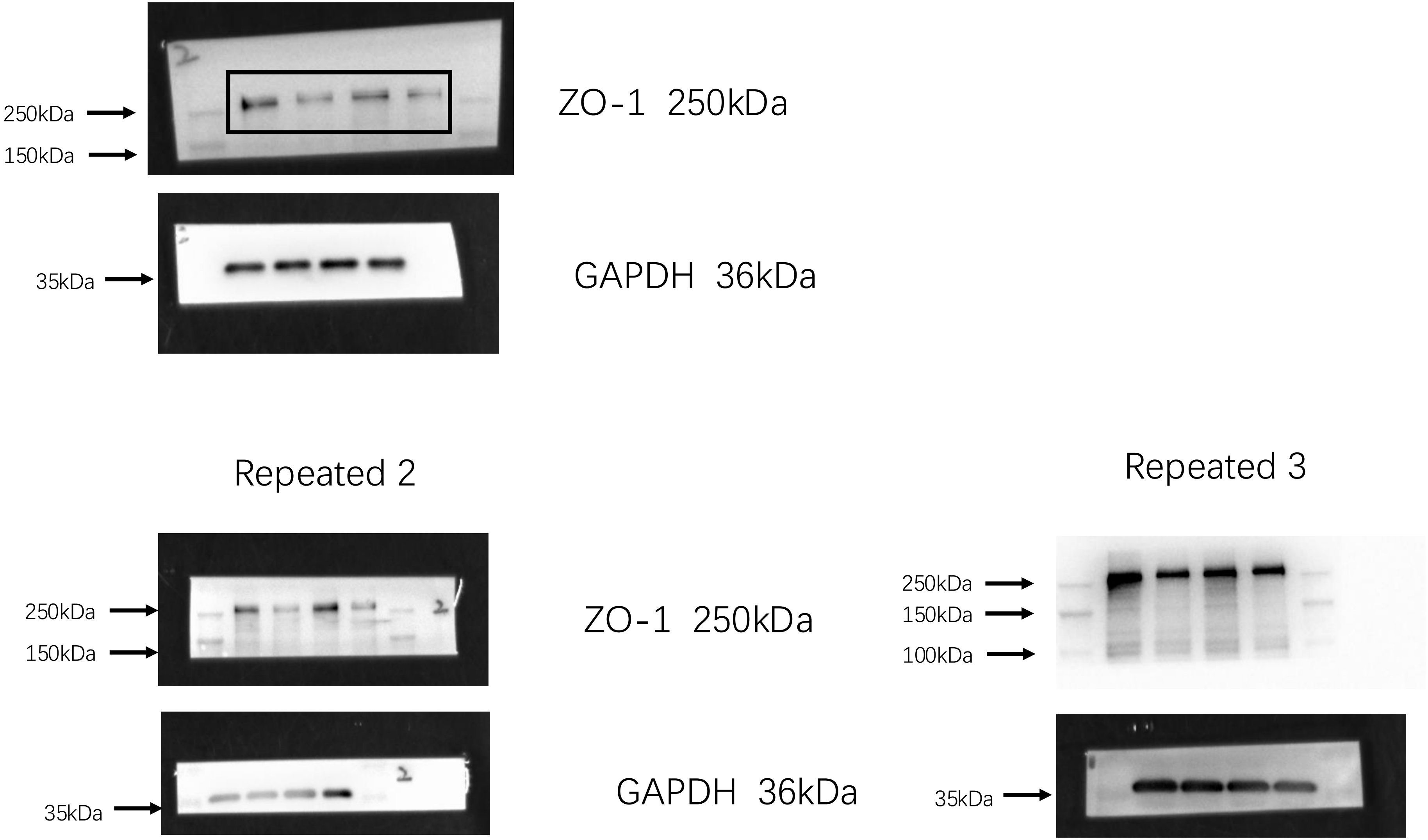

Figure S10. Uncropped and repeated gels for Western blots in Figure 5 of VE-cadherin

150kDa
100kDa

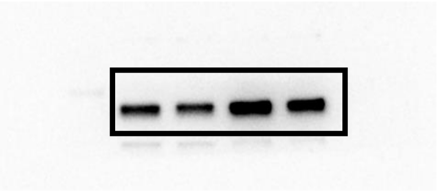

VE-cadherin 130kDa

45kDa
35kDa

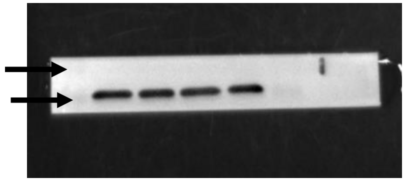

GAPDH 36kDa

Repeated 2

250kDa
150kDa
100kDa

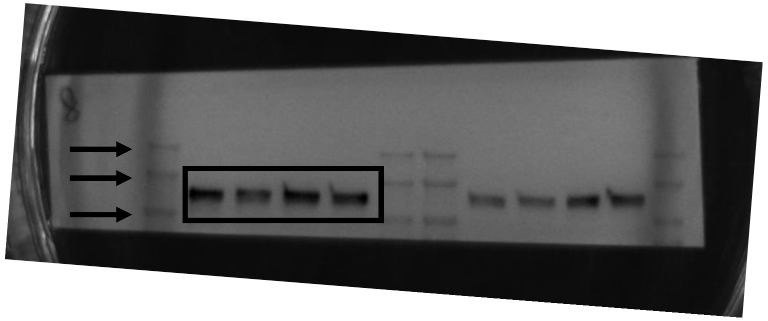

VE-cadherin 130kDa

45kDa
35kDa

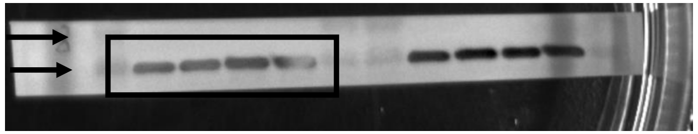

GAPDH 36kDa

Repeated 3

250kDa
150kDa
100kDa

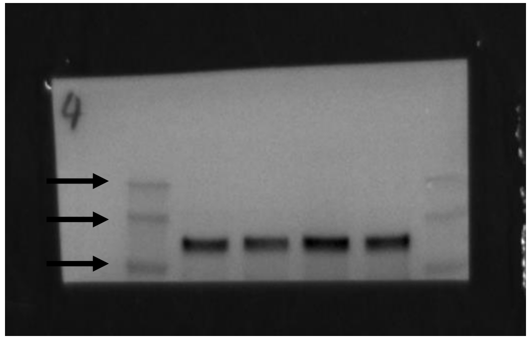

35kDa

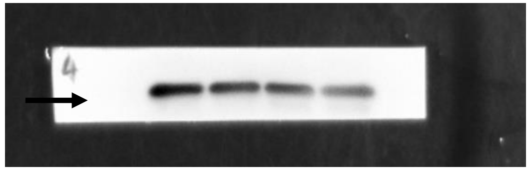

Figure S11. Uncropped and repeated gels for Western blots in Figure 5 of Bcl-2

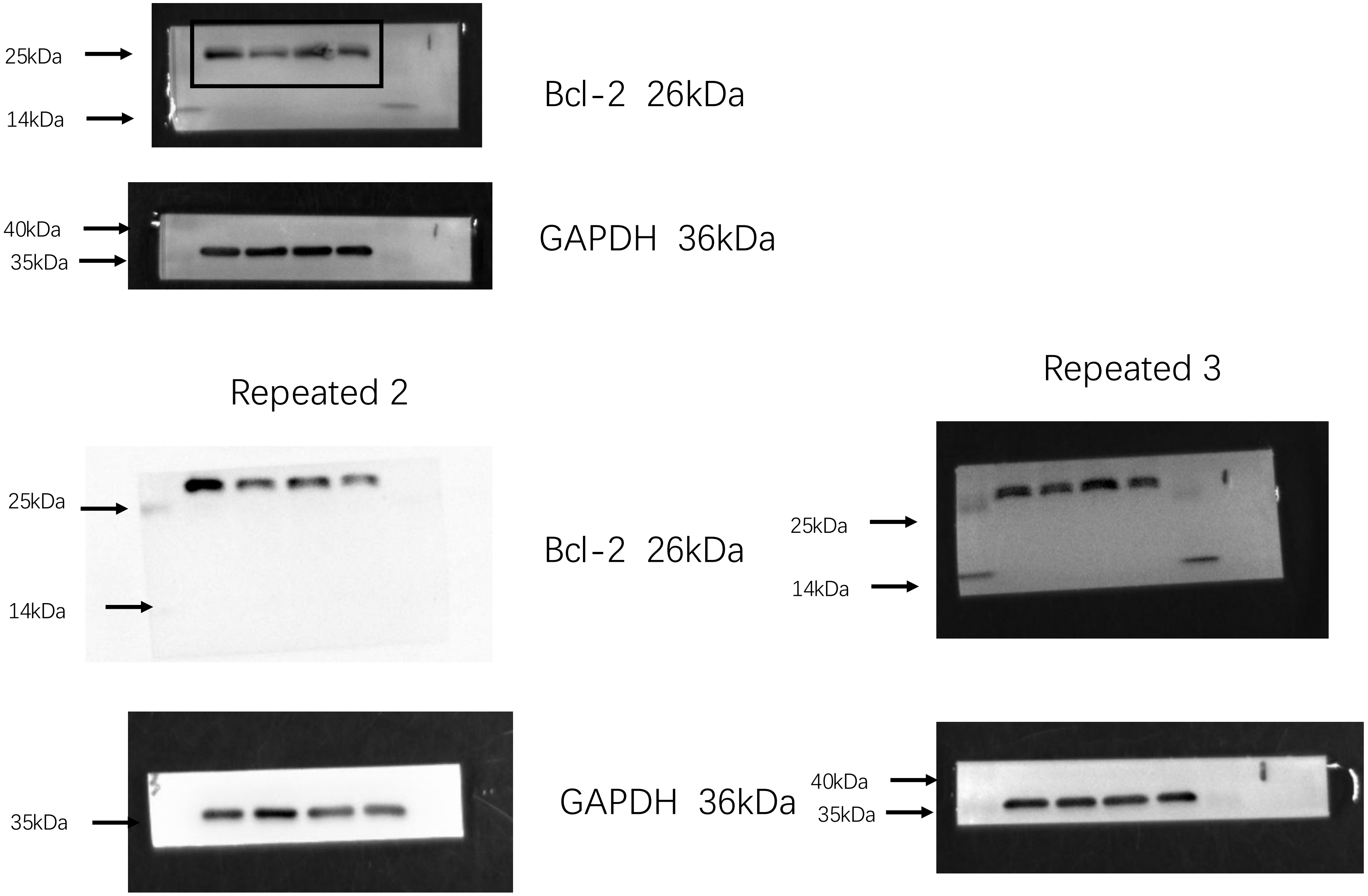

Figure S12. Uncropped and repeated gels for Western blots in Figure 5 of Bax

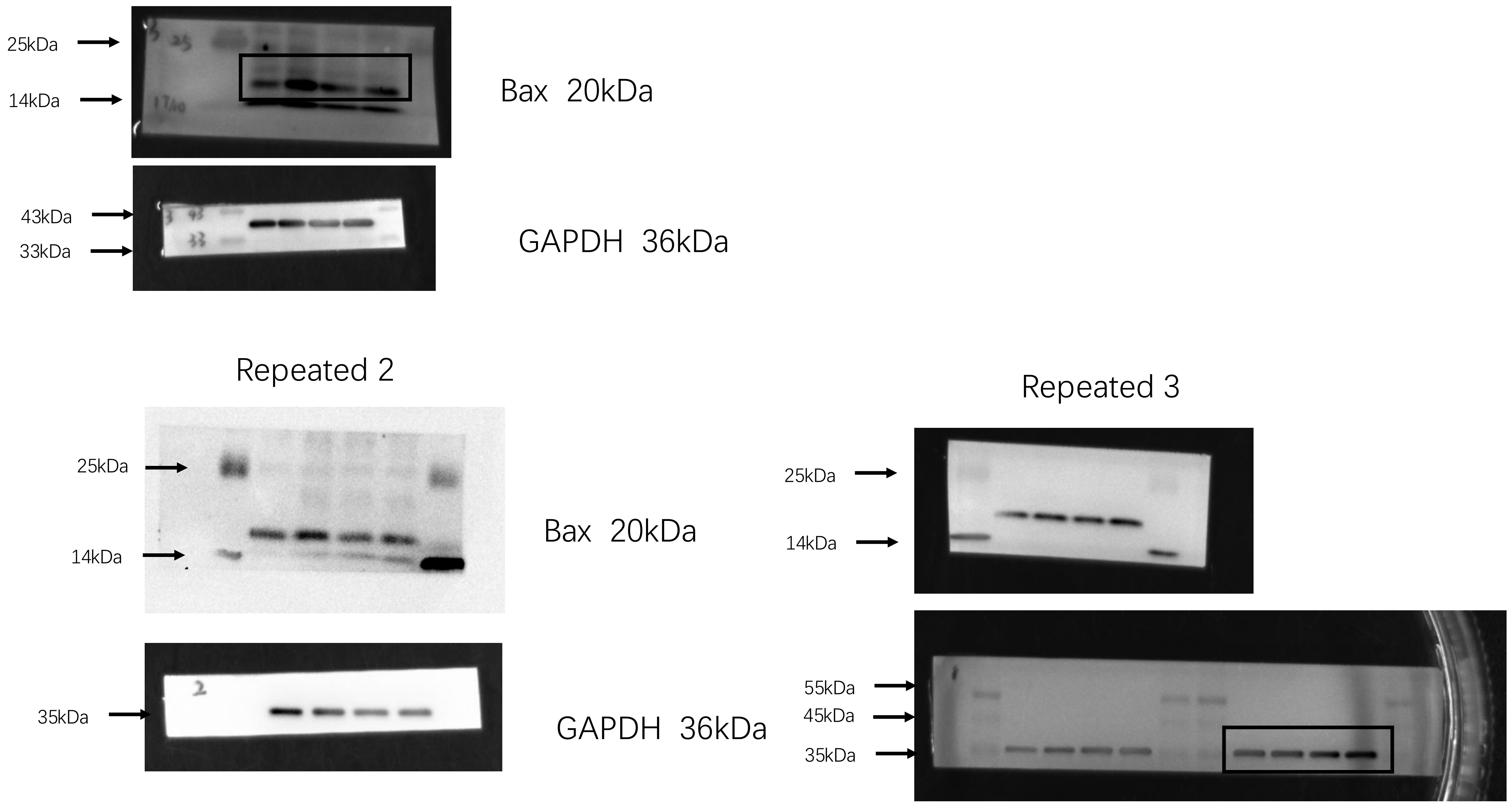

Figure S13. Uncropped and repeated gels for Western blots in Figure 5 of p-PI3K、PI3K

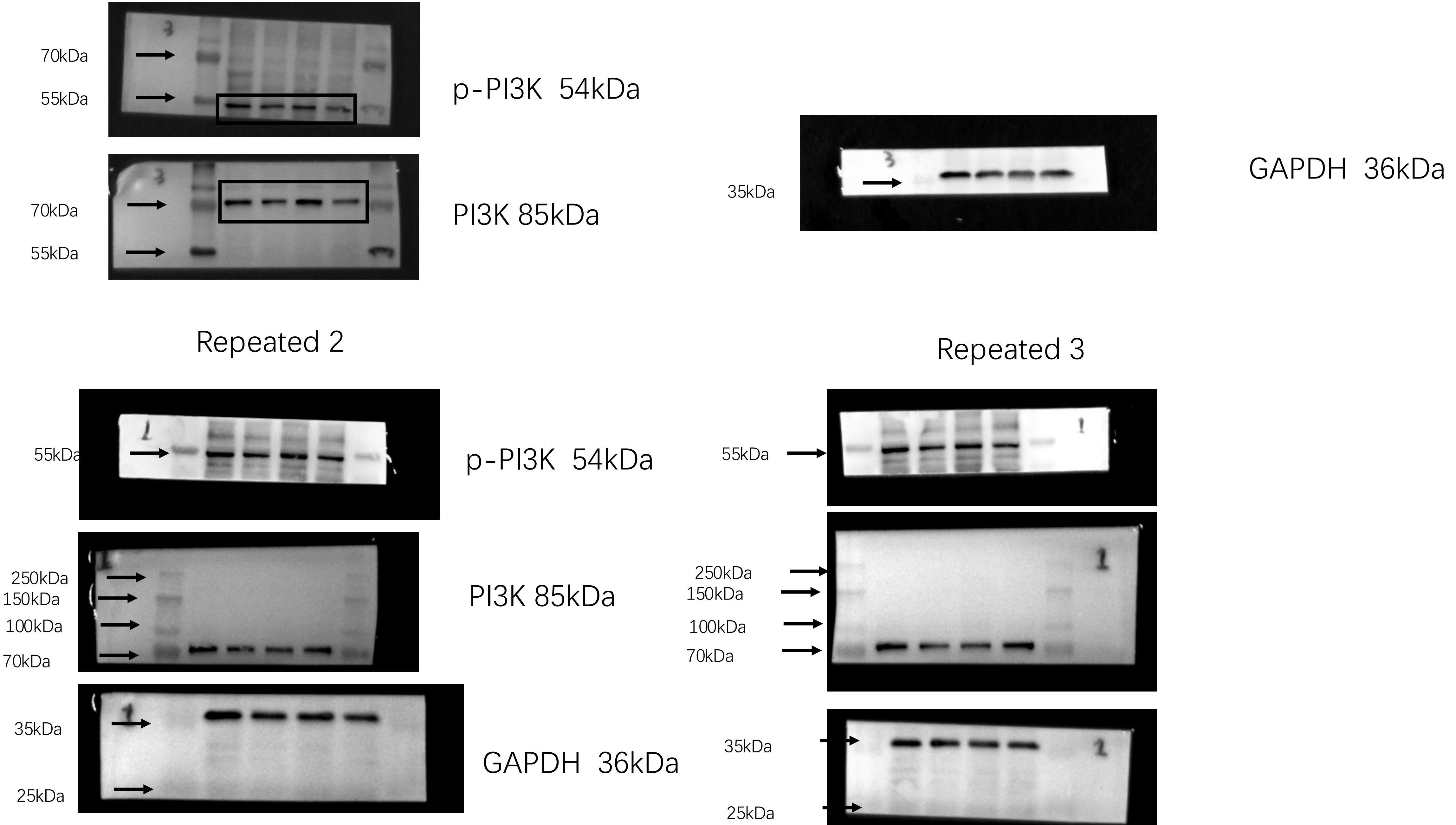

Figure S14. Uncropped and repeated gels for Western blots in Figure 5 of p-AKT、AKT

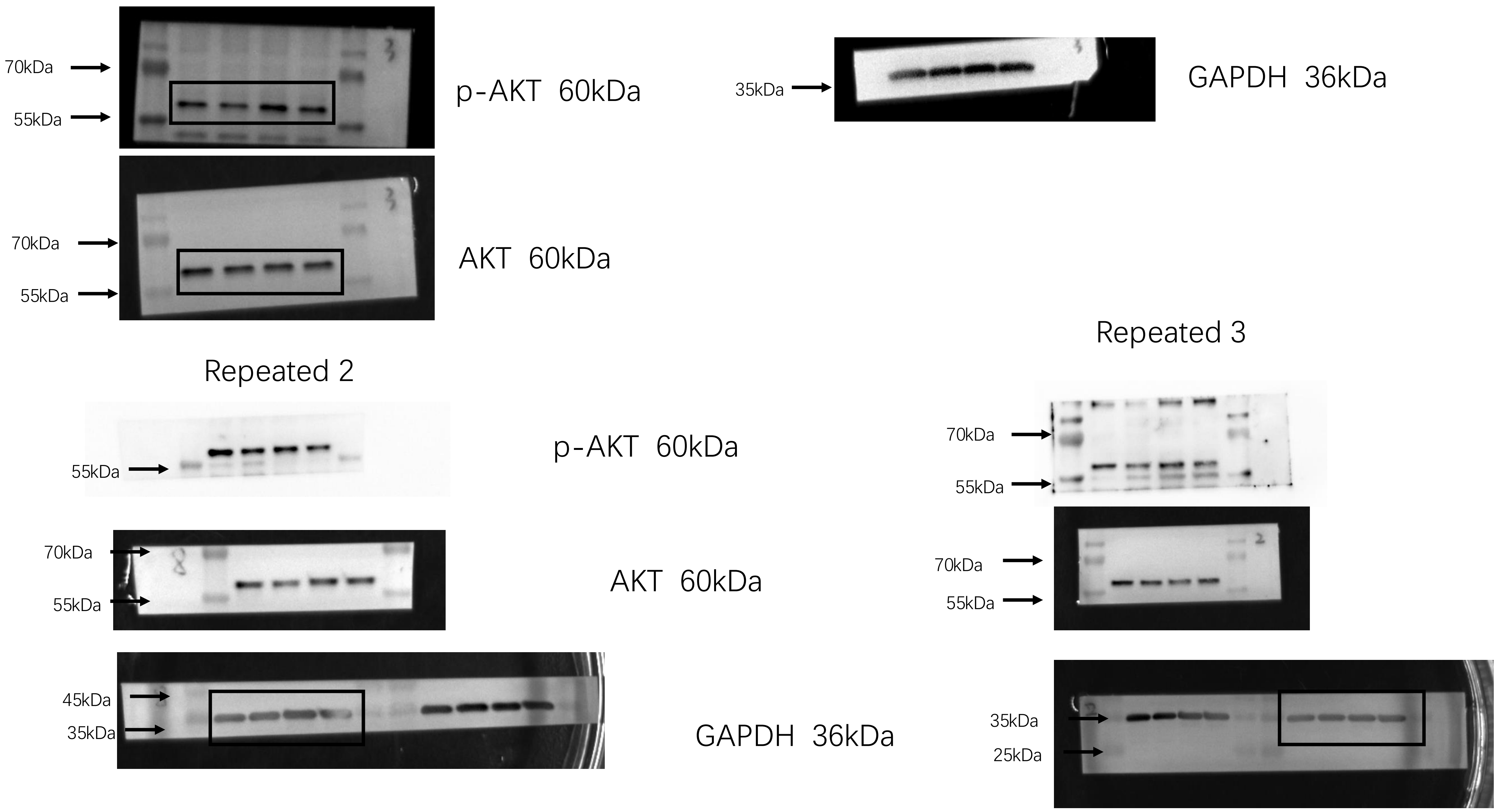

Figure S15. Uncropped and repeated gels for Western blots in Figure 9 of ZO-1

250kDa
150kDa
100kDa

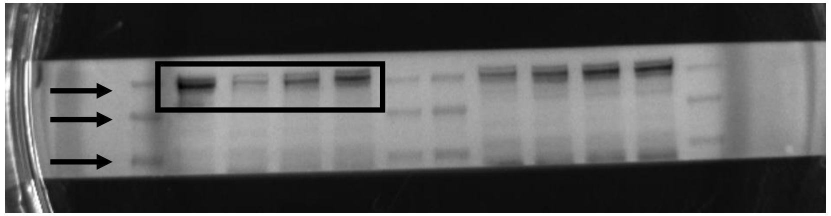

ZO-1 250kDa

45kDa
35kDa

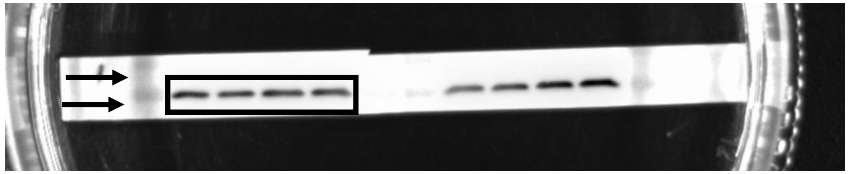

GAPDH 36kDa

Repeated 2

250kDa
150kDa

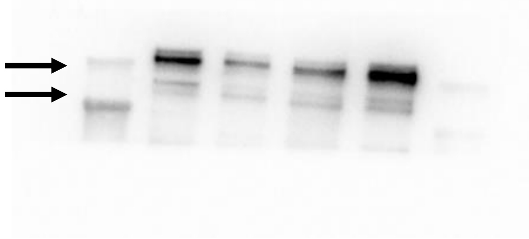

ZO-1 250kDa

Repeated 3

250kDa
150kDa

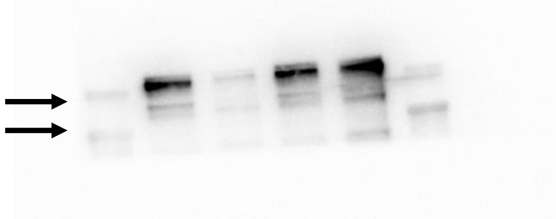

35kDa

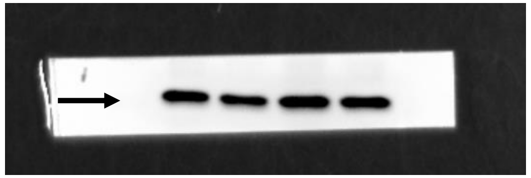

GAPDH 36kDa

35kDa

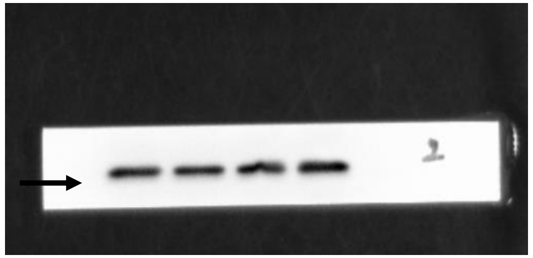

Figure S16. Uncropped and repeated gels for Western blots in Figure 9 of VE-cadherin

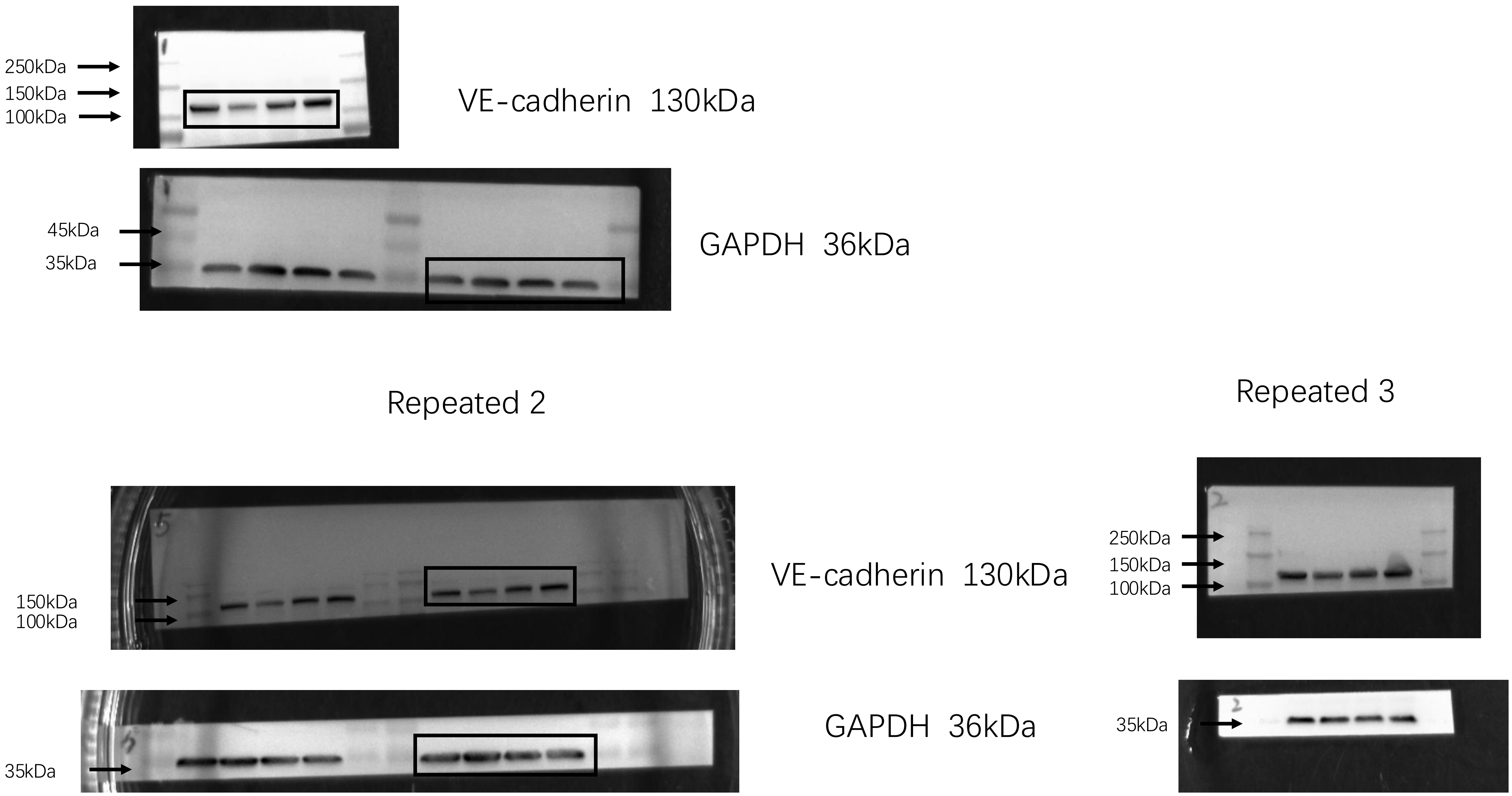

Figure S17. Uncropped and repeated gels for Western blots in Figure 9 of Bcl-2

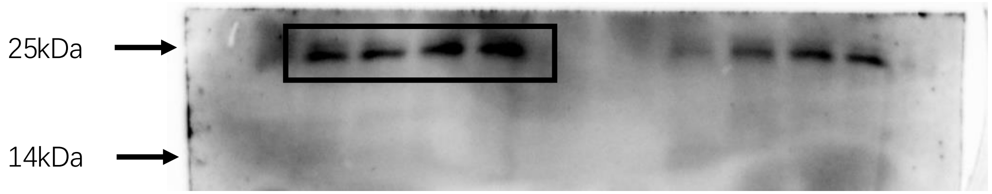

Bcl-2 26kDa

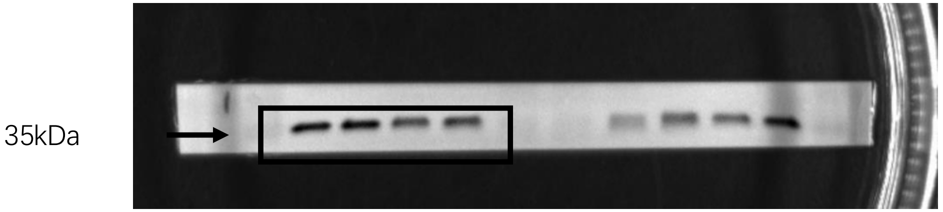

GAPDH 36kDa

Repeated 2

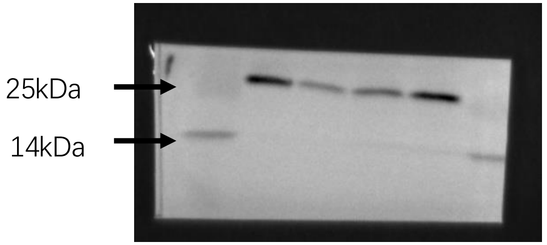

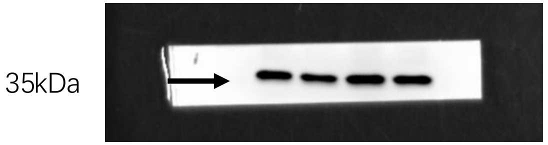

Bcl-2 26kDa

GAPDH 36kDa

Repeated 3

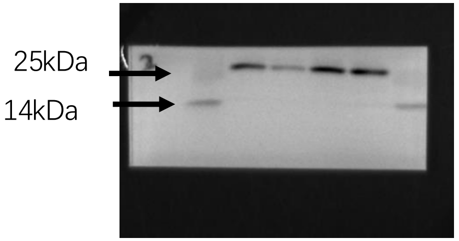

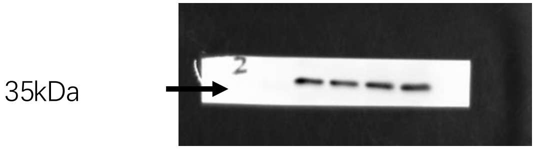

Figure S18. Uncropped and repeated gels for Western blots in Figure 9 of Bax

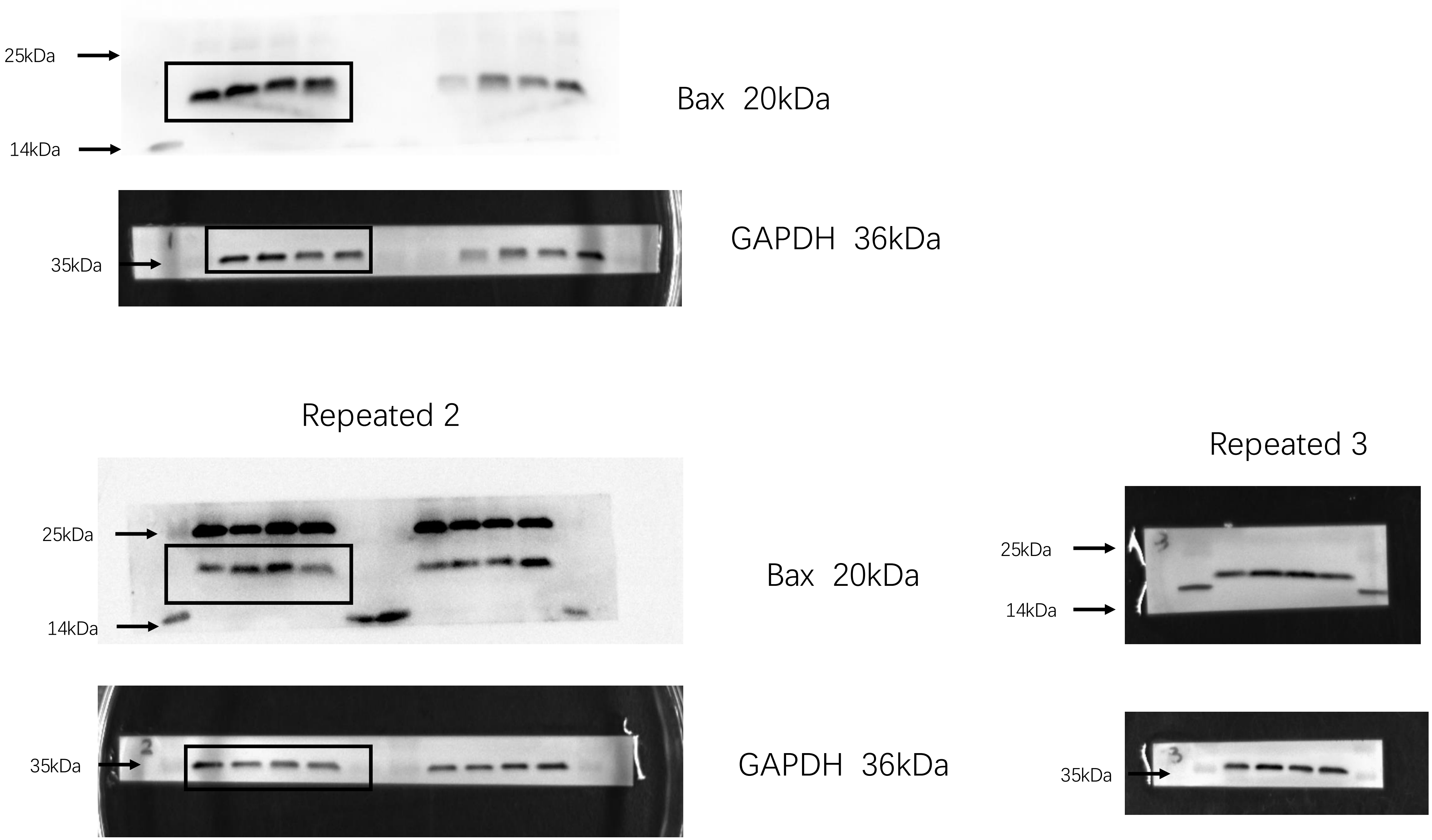

Figure S19. Uncropped and repeated gels for Western blots in Figure 9 of p-PI3K、PI3K

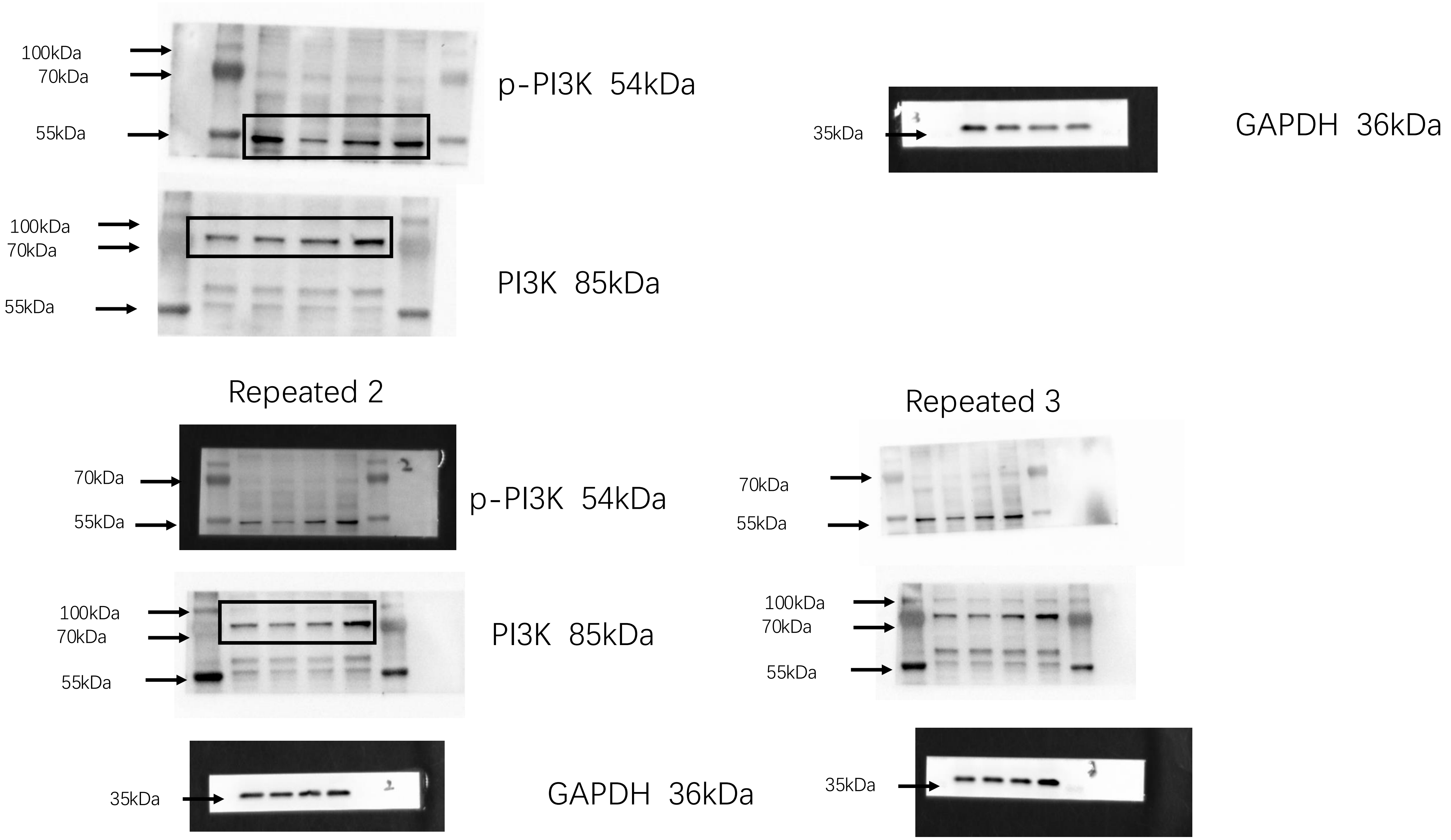

Figure S20. Uncropped and repeated gels for Western blots in Figure 9 of p-AKT、AKT

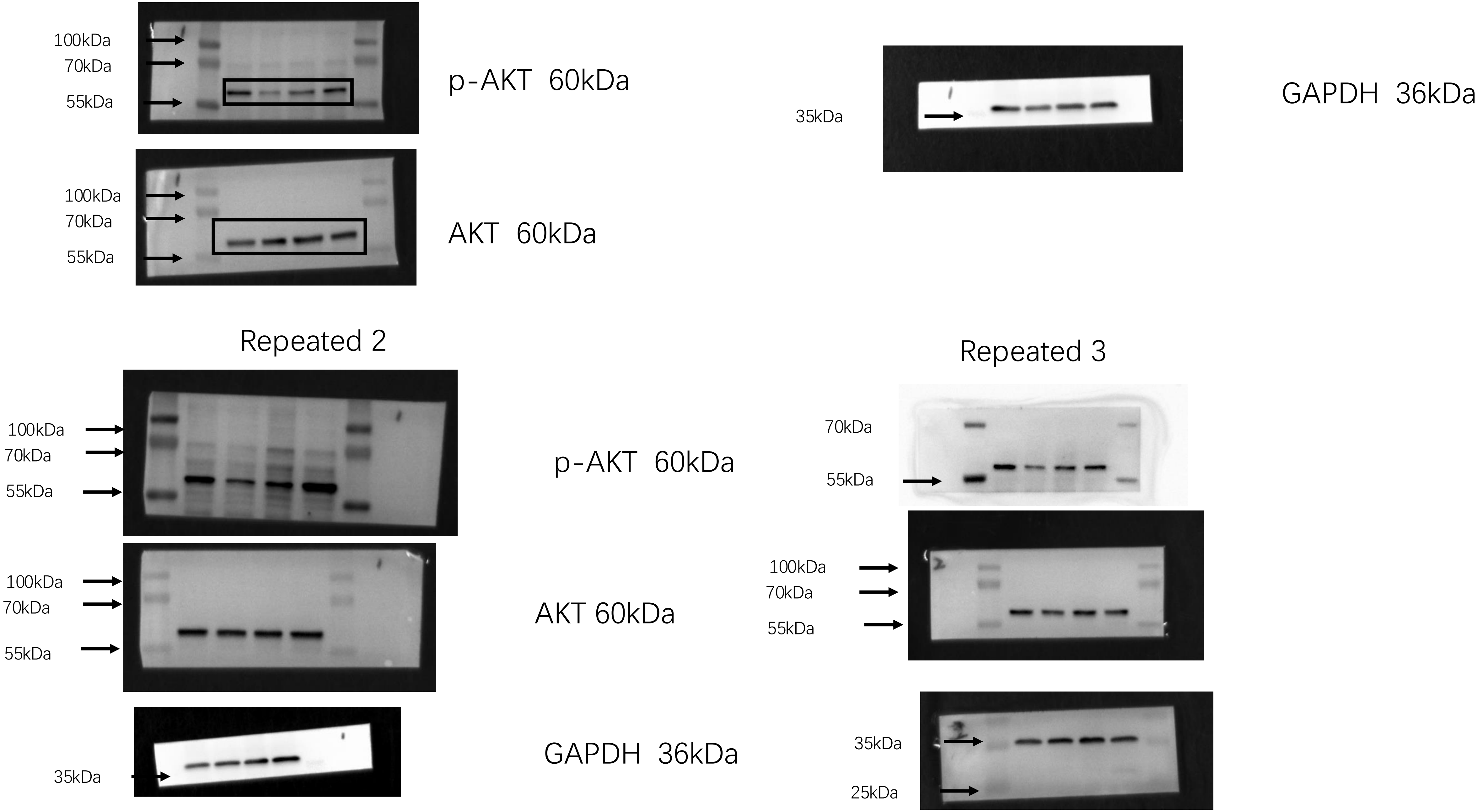

Supplement: Supplementary file 1 [file biomolecules-16-01356-s001.zip › biomolecules-4495071-supplementary.pdf]
